# Strategies for the Production of Soluble Interferon-Alpha *Consensus* and Potential Application in Arboviruses and SARS-CoV-2

**DOI:** 10.3390/life11060460

**Published:** 2021-05-21

**Authors:** Felipe Grabarz, Alexandre Paulo Yague Lopes, Flávia Ferreira Barbosa, Giovana Cappio Barazzone, Jademilson Celestino Santos, Viviane Fongaro Botosso, Soraia Attie Calil Jorge, Ana Lucia Tabet Oller Nascimento, Renato Mancini Astray, Viviane Maimoni Gonçalves

**Affiliations:** 1 Laboratório de Desenvolvimento de Vacinas, Instituto Butantan, São Paulo 05503-900, Brazil; felipe.grabarz@butantan.gov.br (F.G.); alexandre.lopes@butantan.gov.br (A.P.Y.L.); giovana.barazzone@butantan.gov.br (G.C.B.); jademilsonsantos@gmail.com (J.C.S.); ana.nascimento@butantan.gov.br (A.L.T.O.N.); 2 Programa Interunidades em Biotecnologia, Universidade de São Paulo, São Paulo 05508-060, Brazil; flavia.barbosa@butantan.gov.br; 3 Laboratório Multipropósito Viral, Instituto Butantan, São Paulo 05503-900, Brazil; renato.astray@butantan.gov.br; 4 Laboratório de Virologia, Instituto Butantan, São Paulo 05503-900, Brazil; viviane.botosso@butantan.gov.br; 5 Laboratório de Biotecnologia Viral, Instituto Butantan, São Paulo 05503-900, Brazil; soraia.jorge@butantan.gov.br

**Keywords:** antiviral, biopharmaceutical, solubility tag, arboviruses, SARS-CoV-2, COVID-19

## Abstract

Biopharmaceutical production is currently a multibillion-dollar industry with high growth perspectives. The research and development of biologically sourced pharmaceuticals are extremely important and a reality in our current healthcare system. Interferon alpha consensus (cIFN) is a non-natural synthetic antiviral molecule that comprises all the most prevalent amino acids of IFN-α into one consensus protein sequence. For clinical use, cIFN is produced in *E. coli* in the form of inclusion bodies. Here, we describe the use of two solubility tags (Fh8 and DsbC) to improve soluble cIFN production. Furthermore, we analyzed cIFN production in different culture media and temperatures in order to improve biopharmaceutical production. Our results demonstrate that Fh8-cIFN yield was improved when bacteria were cultivated in autoinduction culture medium at 30 °C. After hydrolysis, the recovery of soluble untagged cIFN was 58% from purified Fh8-cIFN molecule, fourfold higher when compared to cIFN recovered from the DsbC-cIFN, which achieved 14% recovery. The biological activity of cIFN was tested on in vitro model of antiviral effect against Zika, Mayaro, Chikungunya and SARS-CoV-2 virus infection in susceptible VERO cells. We show, for the first time, that cIFN has a potent activity against these viruses, being very low amounts of the molecule sufficient to inhibit virus multiplication. Thus, this molecule could be used in a clinical approach to treat Arboviruses and SARS-CoV-2.

## 1. Introduction

Due to many scientific advances, with the use of modern molecular biology tools, the biopharmaceutical industry has become a multibillion-dollar industry. Through the last years, the biotechnology industry invested massively in the production of these molecules indicating a substantial growth dynamic in the future. The “Global Protein Therapeutics Market Outlook 2020” report estimates that by the end of 2020, this market may reach $208 billion dollars [1].

Biopharmaceuticals are characterized as molecules with therapeutic proprieties, produced through biotechnological processes. These drugs are currently used in a wide array of medical specialties and sometimes are considered the most effective treatments. Therapeutic proteins are used in a broad range of diseases, such as cancer, metabolic disorders and immunotherapy [2].

Several biopharmaceuticals are produced in prokaryotic systems, such as the bacteria *Escherichia coli*. The *E. coli* expression system is very attractive for industrial processes as *E. coli* cell molecular biology, uncomplicated manipulation, and easy scale-up are broadly known, favoring target protein production [3,4]. Even though *E. coli* expression host could be considered the best choice for the biotechnology industry, the expression of heterologous proteins still presents many limitations: inadequate disulfide bonds, the presence of endotoxins, chaperone absence, codon incompatibility, and protein aggregation in inclusion bodies. It is estimated that *E. coli* is capable of expressing 75% of human proteins; however, only 25% of these molecules are produced in a soluble and active structure [5,6,7]. As protein solubility is one of the main concerns in recombinant protein production, this work will focus in this particular issue.

Production of soluble proteins in *E. coli* can be increased by fusing molecules (solubility tags) with the target protein. Solubility tags are extremely soluble proteins, or fragments of these proteins, that can also exert chaperone activity. When fused to the protein of interest, they tend to improve folding and protein solubility [8]. Several researchers reported that target proteins were initially produced in inclusion bodies, however, this had been changed after tag insertion, increasing protein solubility [9,10,11,12,13]. Some of the classical solubility tags are MBP (Maltose-binding protein), Trx (Thioredoxin), GST (Glutathione-S-transferase) and SUMO (small ubiquitin-modified) [14]. Fh8 and DsbC are new tags with interesting properties. 

Fh8 (GenBank ID: AF213970.1) is one of the promising tags whose advantage is to provide effective solubility [14,15]. The Fh8 molecule is an antigen of 8 kDa extracted from *Fasciola hepatica* that was initially proposed to be used as a diagnostic tool and vaccine and drug development against the parasite [13]. Fh8 was shown to be an efficient solubility tag to increase the solubility of recombinant *Cryptosporidium parvum* protein (CP12), human interleukin-5 (IL-5) and *Toxoplasma gondii* oocyst wall (TgOWP). These studies reported an increase of 3 to 16 times in production yield when compared to the same proteins without the associated tag [13,16]. Fh8 could also be used as a purification tool when producing recombinant biopharmaceuticals because this tag works as a calcium sensor protein. In the presence of calcium, Fh8 changes its structure and exposes hydrophobic residues, which interact with hydrophobic interaction chromatography resins, such as Phenyl-Sepharose [15].

The formation of disulfide bonds in *E. coli* is mediated by several Dsb family proteins, such as DsbA, DsbB, DsbC, DsbD, DsbE, and DsbG, which assist protein folding [17,18]. DsbC is a soluble protein [19] and works as a dimer displaying two identical subunits which comprises a -Cys^98^-Gly-Tyr-Cys^101^- portion. This amino acid arrangement makes DsbC highly reactive to the disulfide bond, thus assisting protein folding [20]. Even though *E. coli* is widely used to produce recombinant proteins, this expression host is not ideal for disulphide bond formation as *E. coli* cytoplasm exhibit a notably reducing environment favoring protein aggregation [21]; thus, DsbC tag could aid in protein folding, circumventing this issue. The DsbC disulfide isomerase has been described as a solubility tag that prevented formation of inclusion bodies when fused to target proteins containing disulfide bonds [12]. For example, a higher yield (69%) in the production of insulin-like growth factor 1 (IGF-I) was achieved by fusing IGF-I to the DsbC when compared to IGF-I with no tag [22].

A non-natural interferon, also called recombinant interferon-consensus, IFN-con, cIFN or rhIFN-con, was designed following the analysis of the amino acid sequences present in several subtypes of IFN-α [23]. The antiviral activity of cIFN has been shown to be significantly greater than that of IFNα-2α and IFNα-2b due to higher binding avidity of the receptor-protein complex [24,25,26]. As cIFN and IFN type I are mainly produced in inclusion bodies, additional steps in downstream processing are required in order to obtain the molecule in a biologically active form [27,28].

Arboviruses (arthropod-borne viruses) represent a group of viruses that are transmitted by arthropod vectors [29]. Mayaro (MAYV) and Chikungunya (CHIKV) viruses belong to the Togaviridae family and Alphavirus genus. Both viruses are human pathogens causing fever, arthritis, diarrhea, neurologic manifestations and lymphadenopathies [30,31]. Zika virus (ZIKV) belongs to Flaviviridae family, genus Flavivirus. The ZIKV outbreak in the American continent, especially in Brazil from 2015 to 2016, was responsible for congenital Zika syndrome, with mycrocephaly being the most dramatic manifestation. The economic burden of neurological sequelae caused by Zika infection in Latin America can reach billions of USD per year [32]. Although there is a growing focus of scientific research on the studied viruses, several gaps still need to be overcome. There is an urgent need in the development of a prophylactic vaccine and/or antiviral treatment against ZIKV, CHIKV and MAYV [30,31,33].

SARS-CoV-2 belong to the *Coronaviridae* family and *Betacoronavirus* genus [34]. Coronavirus was responsible for at least three epidemic scenarios over the last 20 years: severe acute respiratory syndrome (SARS), Middle Eastern respiratory syndrome (MERS), and currently the COVID-19 disease [35]. The first case of COVID-19 was detected in December 2019 in China and at the time of writing this article there are more than 154 million infections confirmed cases and 3.2 million fatalities [36]. Even though COVID-19 may manifest asymptomatically, the main symptoms are mild to severe respiratory impairment [37], fever, cough [38], pulmonary edema, and acute lung failure [39]. Interestingly, SARS-CoV-2 inhibits the production of IFN-α and blocks cytokine signaling [39].

In this work, cIFN was produced in *E. coli* fused with Fh8 (HF-IFN) or DsbC (HD-IFN) solubility tags and a six-histidine tag (His-tag) at N-terminus of the molecule or exclusively with N-terminal His-tag (H-IFN) and no solubility tag. The HF-IFN recombinant molecule was the best candidate for cIFN production among all analyzed constructs. The HF-IFN protein was produced in the soluble fraction of bacteria when cultivated in autoinduction medium at 30 °C. Additionally, the purified cIFN molecule was tested for antiviral activity against Zika, Chikungunya, Mayaro and SARS-CoV-2 viruses in vitro infection model. The data showed, for the first time, that the cIFN was successfully produced as a soluble molecule and showed strong antiviral activity.

## 2. Materials and Methods

### 2.1. Design and Cloning of HF-IFN, HD-IFN and H-IFN Genes

The sequence of *cifn* gene was based on the article published by Mohammed et al. [28]. Codon optimization was performed with the use of GeneArt™ software (Thermo Fischer Scientific, Waltham, MA, USA). The cIFN nucleotide sequence was submitted to GenBank database (GenBank accession number: MT133905, accessed 3 February 2021). The genetic design of the constructs comprised the inclusion of coding sequences of 6xHis, Fh8 (GenBank ID: AF213970.1) or DsbC (GenBank ID: 947363) tags and tobacco Etch virus (TEV) protease site (ENLYFQ’G recognition region) to the *cifn* gene. *Nco* I and *Xho* I restriction sites were also included flanking regions of interest to allow cloning into pET28a(+) expression vector (Sigma-Aldrich, St. Louis, MI, USA). The *Bam* HI site was incorporated into the 5’ end of the *cifn* gene and 3’ end of the *fh8* or *dsbc* gene sequence. This was accomplished by introducing C671A, A670T, G669C silent mutations in the TEV protease cleavage site. The translation of *6xhis-fh8-cifn* (753 bp) and *6xhis-dsbc-cifn* (1194 bp) gene sequences resulted in the production of HF-IFN and HD-IFN molecules, respectively, and the translation of *6xhis-cifn* (522bp) in the production of H-IFN (Figure S1).

All gene sequences underwent codon optimization and were cloned into pET28a expression vector prior bacterial cultivation. Furthermore, the inserted *Bam* HI restriction site allows substituting the tag or the protein of interest, if necessary.

### 2.2. PCR Amplification

PCR reactions were performed using PCR buffer, 0.2 mM dNTP, 2 mM MgCl_2_, 0.2 mM of each primer (forward and reverse) (Table S1), 2.5 U Taq polymerase (Invitrogen Corporation, Carlsbad, CA, USA), 100 ng DNA, to a final volume of 50 μL. The annealing temperatures used were determined according to the Tm Calculator from Thermo Fischer Scientific (Waltham, MA, USA). Amplification was visualized in 1% agarose gel electrophoresis in TAE buffer (40 mM Tris-acetate and 1 mM EDTA) stained with GelRed™ (Biotium, Inc., Fremont, CA, USA). The amplicons were excised from the gel and purified using the GFxPCR DNA and gel band purification kit (Cytiva, Marlborough, MA, USA).

### 2.3. Bacterial Strains 

*E. coli* DH5α competent bacteria were used for plasmid amplification and molecular cloning. *E. coli* BL21(DE3) was used for gene expression. Both bacterial lineages were obtained from Invitrogen Corporation (Carlsbad, CA, USA).

### 2.4. Culture Media and Conditions

Different culture media were used to evaluate cell growth and protein synthesis. Luria-Broth (LB) [40]: culture medium used to activate frozen stock cells, to evaluate the stability of the plasmid (Table S2a) and to compare the production of H-IFN and HF-IFN. Autoinduction (AI): formulation of the autoinduction complex medium (Table S2b) is based on the ZYM-5052 medium developed by Studier [41] and modified by Dos Santos [42]. SDAB: chemically defined autoinduction medium [43] (Table S2c). HDF: chemically defined medium [44] (Table S2d). Bacterial growth was performed in Erlenmeyer or TunAir™ flasks (Z710822, Sigma-Aldrich, St. Louis, MI, USA), at 25 °C or 30 °C at 300 RPM agitation. Protein expression was induced with 0.1 mM IPTG or lactose at 25 °C or 30 °C.

To compare the solubility with and without Fh8 tag, *E. coli* BL21(DE3) transformed with plasmid pET28a-*hf-ifn* or pET28a-*h-ifn* were cultured at 30 °C in Erlenmeyer flasks (1 L) with 200 mL of LB medium and induced with 0.1 mM IPTG when OD = 0.6. Samples from soluble and insoluble fractions were analyzed before and up to 6 h after IPTG induction.

To evaluate HF-IFN production and solubility, *E. coli* BL21(DE3) transformed with plasmid pET28a-*hf-ifn* was cultured at 25 °C or 30 °C in TunAir™ 300 mL-flasks containing 100 mL of medium and induced with 0.1 mM IPTG when OD600nm = 0.6 in HDF medium or lactose when glucose was exhausted in SDAB and autoinduction media. Samples of soluble fractions were analyzed before and up to 10 h after IPTG induction for HDF medium, and after glucose consumption for autoinduction and SDAB media, respectively, 4 to 5 h and 7 to 10 h after the beginning of cultivation. The same procedure was employed to evaluate HD-IFN production and solubility.

### 2.5. Evaluation of Protein Synthesis

Protein synthesis was evaluated by sodium dodecyl sulfate polyacrylamide gel electrophoresis (SDS-PAGE), 15% gels. Samples with a volume equivalent to optical density (OD600nm) equal to 5.0 were withdrawn from the cultures and centrifuged at 15,000× *g* for 5 min (Centrifuge 5424R, Eppendorf, Hamburg, Germany). The pellets were resuspended in 500 μL of saline and samples were sonicated for 2 min with pulses of 15 s on/off by the Branson Ultrasonic Sonifier S-250D (Brookfield, CO, USA). After sonication, samples were centrifuged at 15,000× *g* for 30 min and the soluble and insoluble fractions were separated. The pellet was resuspended in 80 μL of MilliQ water and 20 μL of SDS-PAGE 5× sample buffer. To analyze the soluble fraction, 80 μL of the supernatant was mixed with 20 μL of the 5× sample buffer. Samples were loaded into 15% polyacrylamide gel after heating samples at 96 °C for 5 min. The gels were stained with Coomassie Blue R-250 (Sigma-Aldrich, St. Louis, MI, USA). 

### 2.6. Purification of Recombinant H-, HF-IFN and HD-IFN

The bacterial culture was centrifuged at 5000× *g* for 30 min and the supernatant was discarded. The pellet was resuspended in lysis buffer (20 mM phosphate buffer pH 7.4, 500 mM NaCl, 1 mM PMSF, 0.1% Triton X-100). Bacterial cells were disrupted by high-pressure homogenizer (PANDA Plus 2000, GEA, Düsseldorf, Germany) through three passages at 1200 bar, keeping the cell suspension in an ice bath during the passages. All purification methods were conducted using 15 to 20 g of bacterial biomass.

IMAC: The chromatography column (XK26) was packed with 50 mL IMAC-Sepharose 6 Fast Flow resin (Cytiva, Marlborough, MA, USA), the resin was charged with Ni^2+^ and chromatography was performed on ÄKTA Avant (Cytiva, Marlborough, MA, USA). The column was previously equilibrated with a buffer containing 20 mM phosphate, pH 7.4, 500 mM NaCl buffer and 10 mM imidazole. Elution was performed with increasing concentrations of imidazole (50 mM, 150 mM, and 500 mM).

Q-Sepharose: The chromatography column (XK26) was packed with 74 mL of Q-Sepharose Fast Flow (Cytiva, Marlborough, MA, USA). The column was previously equilibrated with 50 mM Tris-HCl, pH 8.0. Elution was performed with increasing concentrations of NaCl (0 to 0.7 M).

### 2.7. Tag Removal 

The fusion proteins encompass a TEV protease hydrolysis site, which is located between C-terminal end of the Fh8 or DsbC and N-terminal end of the consensus interferon. Thus, the TEV protease hydrolysis generates two protein fragments. The first fragment refers to the Fh8 or DsbC tag associated with the N-terminal histidine tag, which have molecular mass of 9.4 kDa and 24.2 kDa, respectively. The second protein fragment, containing the biopharmaceutical product of interest cIFN, has a theoretical molecular mass of 19.4 kDa (Figure S10).

After Q-Sepharose and IMAC, HF- and HD-IFN samples were dialyzed against 1 L of phosphate buffered saline (PBS), PBS was changed three times every 1 h for removal of the imidazole. Then, the solubility tags were removed by hydrolysis with TEV protease (Cellco Biotec, São Carlos, São Paulo, Brazil). The hydrolysis reaction was conducted overnight using a 1:100 ratio (enzyme mass: protein mass) in 25 mM Tris-HCl, pH 8.0, 150 mM NaCl, and 14 mM β-mercaptoethanol. In order to remove the hydrolyzed products, a HiTrap IMAC Sepharose FF column (Cytiva, Marlborough, MA, USA) was used. The column was equilibrated with 25 mM Tris-HCl pH 7.4 with 500 mM NaCl and 10 mM imidazole. The cleaved cIFN fraction was collected in the non-adsorbed fraction. Fh8 and DsbC solubility tags and TEV protease, which have a 6xHis tag in their N-terminal portion, as well as non-hydrolyzed product, were eluted with 500 mM imidazole.

### 2.8. Purity Determination and Quantification of Target Protein by Densitometry

Protein purity was quantified by densitometry of SDS-PAGE bands using the BioRad GS-800 densitometer and Quantity One 4.6.3 software (BioRad, Hercules, CA, USA). The relative percentage of the target protein band was calculated by Equation (1):

relative quantity (%) = (target protein band intensity × 100)/(∑all bands intensity (same lane))(1)

Total protein concentration was measured by the DC Protein Assay Lowry method (BioRad, Hercules, CA, USA) and the concentration of the target protein was calculated using the values of relative quantity according to the Equation (2):

(2) $$\mathrm{target}\;\left(\mathrm{mg}/\mathrm{mL}\right)=\frac{\mathrm{relative}\;\mathrm{quantity}\;\left(\%\right)\times \;\mathrm{protein}\;\mathrm{concentration}}{100}$$

### 2.9. High-Performance Size-Exclusion Chromatography (HPSEC)

Purity determination of cIFN was performed by Agilent 1260 Infinity equipment (Agilent, Santa Clara, CA, USA). Sample and mobile phase were filtered at 0.22 µm. The TSKGel pre-column (PW-Type) and the HPLC column TSKGel G3000 PWXL (Sigma-Aldrich, St. Louis, MI, USA) were used. The mobile phase is composed of 10 mM sodium phosphate, pH 7.5 and 150 mM NaCl. The flow rate used was 0.4 mL/min with a final injection volume of 20 µL. The protein peaks were detected by UV at 280 nm.

### 2.10. Antiviral Activity of cIFN

Vero cells (ATCC® CCL-81™, Manassas, VA, USA) were cultured in D-MEM (Thermo Scientific, Waltham, MA, USA) with 10 % Fetal Calf Serum (FCS, Sigma-Aldrich, St. Louis, MI, USA) and incubated at 37 °C with 5% CO_2_ atmosphere in all the different steps of the antiviral test. Zika virus (ZIKV, BeH815744) was kindly provided by Dr Pedro Vasconcelos (Evandro Chagas Institute), Mayaro virus (MAYV, BeAr20290) was kindly provided by Dr Maurício Nogueira (São José do Rio Preto School of Medicine), Chikungunya virus (CHIKV) was isolated from a serum sample taken during an outbreak in Sergipe, Brazil, 2017, which was kindly provided by Dr Alessandra Schanoski (Bacteriology Laboratory, Butantan Institute) and SARS-CoV-2 (SARS-Cov2/SP02/2020HIAE) was isolated from a nasopharyngeal swab and kindly provided by Dr Edison Luis Durigon (ICB, University of São Paulo).

Each antiviral test was performed in three different experiments with three analytical triplicates each. Firstly, cells were seeded in 96-well plates (1 × 10^4^ cels/well) and grown for 48 hours until they reached 80% confluence. A sample corresponding to the cells of three wells was obtained after trypsinization and used for cell counting. Culture medium was replaced by serially diluted cIFN in new culture medium, starting at 640 pg/mL (SARS-CoV-2), 160 pg/mL (for CHIKV and MAYV) or 2.67 pg/mL (for ZIKV), and cells were incubated for two hours. Cells were then infected with 0.1 MOI of one of the arboviruses in study or 0.02 MOI for SARS-CoV-2. Controls (not infected cells or infected cells) were used for their respective viruses. Plates were incubated and a cytopathic effect was observed after 48 h for MAYV, 72 h for CHIKV and SARS-CoV-2, and 96 h for ZIKV. Following this, the culture medium was removed, plates were washed with PBS (pH 7.2), and staining was performed with NBB Solution (0.1% Naftol Blue Black, 1.6% Sodium acetate, 6% acetic acid) for 30 min. The staining solution was removed, the plates were rinsed with water and the absorbance was measured at a wavelength of 450 nm in a microplate reader (Original Multiskan EX, Thermo Scientific, MA, USA). Mean absorbance (abs) values of each cIFN dilution, and infected and non-infected control analytical triplicates were used for the calculation of the infection inhibition rate for each experiment, defined by the Equation (3):

(3) $$\%\;\mathrm{Inhibition}=\frac{\mathrm{Mean}\;\mathrm{abs}\;\mathrm{cIFN}\;\mathrm{dilution}-\mathrm{Mean}\;\mathrm{abs}\;\mathrm{infected}\;\mathrm{cells}\;\mathrm{control}}{\mathrm{Mean}\;\mathrm{abs}\;\mathrm{non}\;\mathrm{infected}\;\mathrm{cells}\;\mathrm{control}-\mathrm{Mean}\;\mathrm{abs}\;\mathrm{infected}\;\mathrm{cells}\;\mathrm{control}}\times 100$$

Inhibition rates were plotted against respective cIFN concentration and the resulting curve was used for the calculation of EC50 (effective concentration inhibiting 50% of infection). The three EC50 values obtained for each virus tested were expressed as mean ± standard deviation. The cIFN used in this assay was purified from the HF-IFN construction.

## 3. Results

### 3.1. Consensus IFN and Fusion with Solubility Tags

**Figure 1:**
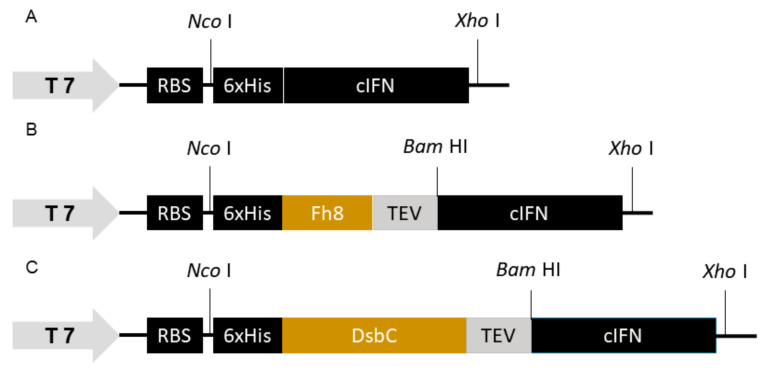
Overview of genetic constructs cloned into pET28a(+) expression vector. (**A**) H-IFN: the gene sequence for coding 6xHis tag and the cIFN; (**B**) HF-IFN: *cifn* gene fused with *Fh8* gene; (**C**) HD-IFN: *cifn* gene fused with *dsbC* gene. T7: RNA polymerase promoter, RBS: Ribosome-binding site, TEV: TEV protease cleavage site. *Nco* I, *Xho* I and *Bam* HI refer to enzyme restriction sites used for cloning strategies in the different protein constructs.

Three variants of the cIFN fused to 6xHis, 6xHis-Fh8 or 6xHis-DsbC tags were constructed (Table 1 and Figure 1). The *cifn* and *fh8* gene sequences were synthetized fused with the *6xhis-tag* sequence for generating the *hf-ifn* insert. This construct served as template for a PCR reaction in order to amplify the *cifn* gene and to clone it to create the *6xhis-cifn* (*h-ifn*) insert, i.e., the construct without the solubility tag. The *dsbC* gene sequence was synthesized and amplified by PCR. The *dsbC* insert was cloned into *Nco* I and *Bam* HI sites of pET28a-*hf-ifn*. This procedure allowed for the removal of the *fh8* cassette and insertion of *dsbC* gene and resulted in the *6xhis-dsbC-cifn* (*hd-ifn*) construct. The resulting plasmid was named pET28a-*hd-ifn*.

Figures related to the amplification, digestion and visualization of bands of interest are presented in Figures S2–S7.

### 3.2. The Presence of the Fh8 Tag Increases HF-IFN Solubility

In order to compare the solubility in the presence or absence of the Fh8 tag, two protein constructs were used: HF-IFN, which has the Fh8 tag fused to cIFN and H-IFN, which is not embedded with the solubility tag (Table 1).

**Figure 2:**
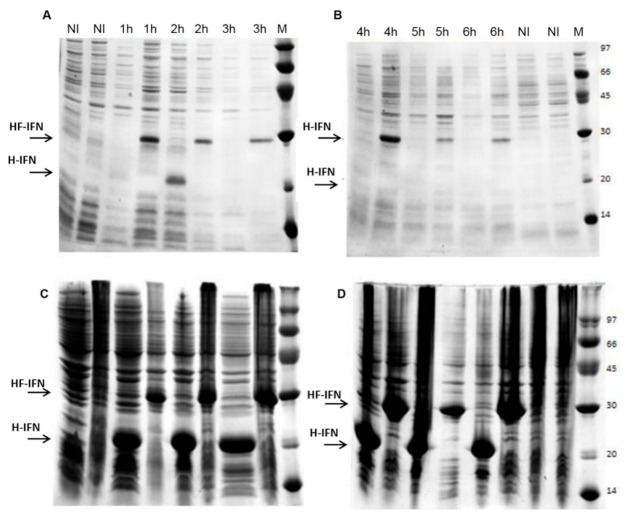
Analysis of H-IFN and HF-IFN production in LB medium by SDS-PAGE. (**A**,**B**): soluble fractions; (**C**,**D**): the insoluble fractions. SDS-PAGE gel lanes alternate between H-IFN (the first lane in each gel) and HF-IFN (the second lane in each gel). (M) molecular marker (kDa); (NI) non-induced. The numbers above the lanes indicate the time elapsed after induction with IPTG. The arrows indicate the size of the bands of interest: HF-IFN 28.8 kDa and H-IFN 20.2 kDa.

The evaluation of the results of gene expression shows that in the absence of Fh8, cIFN tends to aggregate and form inclusion bodies. H-IFN was detected in the soluble fraction only in the sample harvested 2 h after IPTG induction (Figure 2A) and accumulated in inclusion bodies thereafter (Figure 2C,D). The presence of Fh8 improved the molecule solubility when compared to the H-IFN construct. In this culture, after 1 h of IPTG induction, the protein was already observed in the soluble fraction (Figure 2A), remaining soluble up to 6 h after induction with IPTG (Figure 2B). Thus, the results indicated that the Fh8 tag has a good potential for augmenting cIFN solubility in *E. coli*.

The relative amount of target protein was 20% for the two constructs analyzed after 2 h of IPTG induction (Figure S8). It is interesting to note that after this point, the H-IFN construct aggregated in the insoluble inclusion bodies, until the end of the culture (Figure 2). Although HF-IFN construct was also present in the inclusion bodies (Figure 2C,D), part of it remained in the soluble fraction until the end of cultivation, displaying a peak of 27% relative amount after 4 h of induction (Figure S8).

### 3.3. HF-IFN Production Is Higher When Produced in Autoinduction Medium at 30 °C

**Figure 3:**
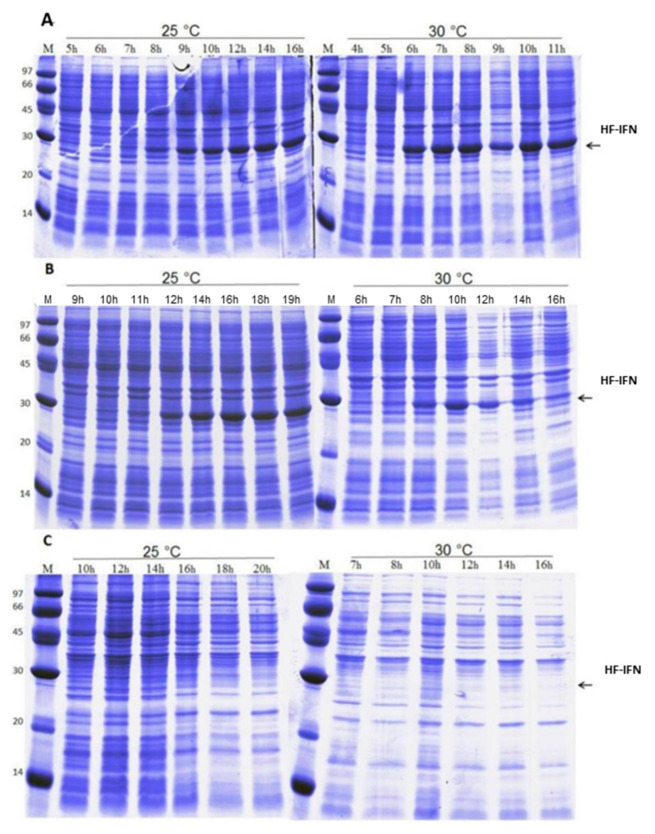
Analysis of HF-IFN production in autoinduction (**A**), HDF (**B**) and SDAB (**C**) culture media. (M) molecular marker (kDa). The numbers above the lanes indicate the total culture time. Arrows indicate the size of the band of interest: HF-IFN 28.8 kDa.

**Figure 4:**
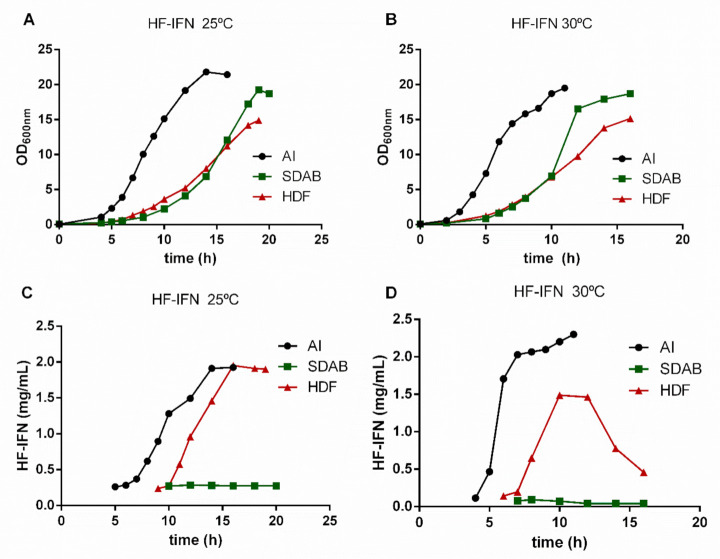
Growth curves (**A**,**B**) and concentration of HF-IFN (**C**,**D**) evaluation in different culture media and temperatures. The concentration of the target protein was determined by densitometry (Equation (2)).

In order to determine the most suitable culture medium to increase the production scale, three different culture media were tested: two chemically defined (HDF and SDAB) and a complex medium (autoinduction medium). The results demonstrated that HDF and autoinduction media were superior when compared to the SDAB culture medium. The culture of *E. coli* for producing HF-IFN (Figure 3 and Figure 4) reached maximal production of the target protein (2.52 g/L) in autoinduction medium at 30 °C (Figure 4D). After 11 h from the start of culture, OD600nm was 19.5 in TunAir flasks (Figure 4B), a 2.2-fold higher value when compared to Erlenmeyer cultivation (data not shown).

### 3.4. HD-IFN Production Is Higher When Produced in Chemically Defined Medium at 25 °C

**Figure 5:**
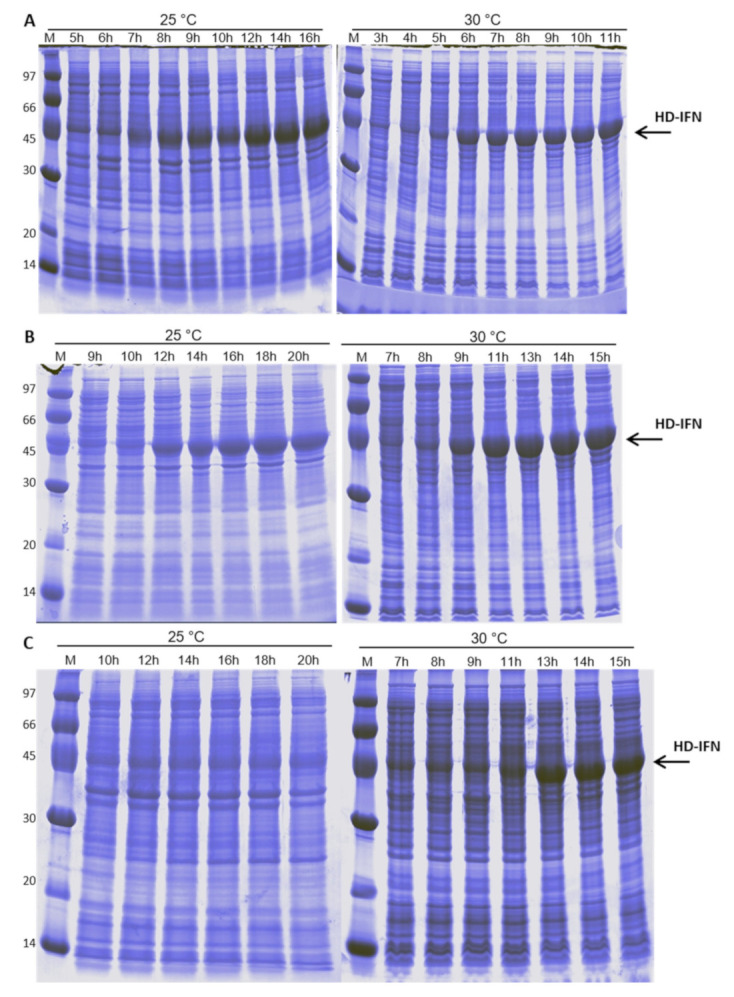
Analysis of HD-IFN production in autoinduction (**A**), HDF (**B**) and SDAB (**C**) culture media. (M) molecular marker (kDa). The numbers above the lanes indicate the total culture time. Arrows indicate the size of the band of interest: HD-IFN 44.6 kDa.

**Figure 6:**
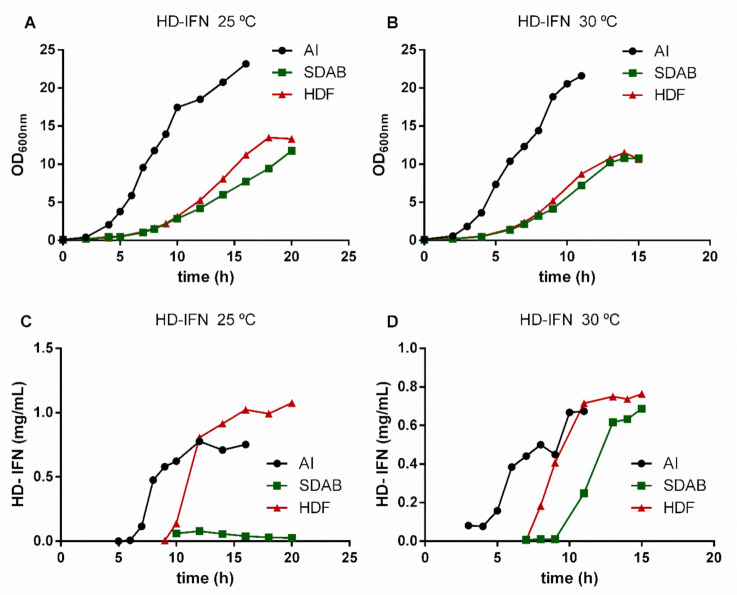
Growth curves (**A**,**B**) and concentration of HD-IFN (**C**,**D**) measurements in different culture media and temperatures. The concentration of the target protein was determined by densitometry (Equation (2)).

*E. coli* culture containing the pET28a-*hd-ifn* plasmid reached maximal production of the target protein in HDF medium at 25 °C after 11 h of induction with 0.1 mM IPTG (Figure 5B and Figure 6C). The maximum concentration of HD-IFN was 1.07 g/L and the OD600nm at the same time was 13.3 (Figure 6A).

When comparing all data obtained, the results showed that the best candidate for bioprocess upscale was the HF-IFN construct in autoinduction medium, and the bacterial culture conducted at 30 °C (Figure S9). The second option would be the HD-IFN construct in HDF medium at 25 °C, because a chemically defined medium, such as HDF, would be preferable to a complex medium, if it yielded a similar amount of the target protein.

### 3.5. Purification of Recombinant cIFN with or without Solubility Tag

#### 3.5.1. Purification of H- and HD-IFN

**Figure 7:**
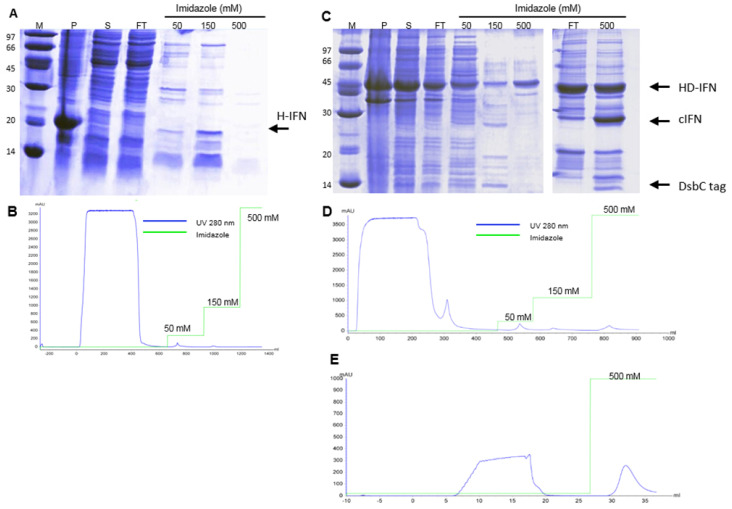
Purification of H- and HD-IFN proteins by IMAC-Ni^2+^ before and after tag removal. (**A**,**C**) SDS-PAGE of fractions collected from H- and HD-IFN purification, respectively. (**B**,**D**) chromatograms of H- and HD-IFN purification, respectively. (**E**) Chromatogram representing the separation of free cIFN (flowthrough), non-hydrolyzed proteins, TEV protease and hydrolyzed solubility tag (500 mM imidazole). (M) Molecular marker, (P) pellet after cell lysis (insoluble fraction), (S) soluble fraction, (FT) flowthrough. H-IFN has 20.2 kDa, HD-IFN has a 44.6 kDa and cIFN has a 19.4 kDa.

All recombinant molecules used in this study comprise a 6xHis-tag. In order to purify the H- and HD-IFN constructs, a metal affinity purification step was performed using IMAC-Sepharose FF loaded with Ni^2+^. Most of the H-IFN produced was aggregated in inclusion bodies (Figure 7A), and a very low amount of H-IFN was detected in the purification fractions analyzed (Figure 7A), despite very small elution peaks being observed in IMAC-Ni^2+^ chromatogram (Figure 7B). Conversely, the HD-IFN was eluted with 150 mM imidazole, reaching 22.2 mg of target protein (Figure 7C,D).

Although the hydrolyzed cIFN from HD-IFN construct was recovered in the flowthrough fraction of IMAC2 (Figure 7C,E), the molecule was not pure. Furthermore, we also noticed that a significant amount of non-hydrolyzed HD-IFN was recovered when eluted with 500 mM imidazole. This finding may reflect the incapability of TEV protease to reach the cleavage site in this particular molecule, which diminishes cIFN yield (Figure 7C).

#### 3.5.2. Purification of HF-IFN

**Figure 8:**
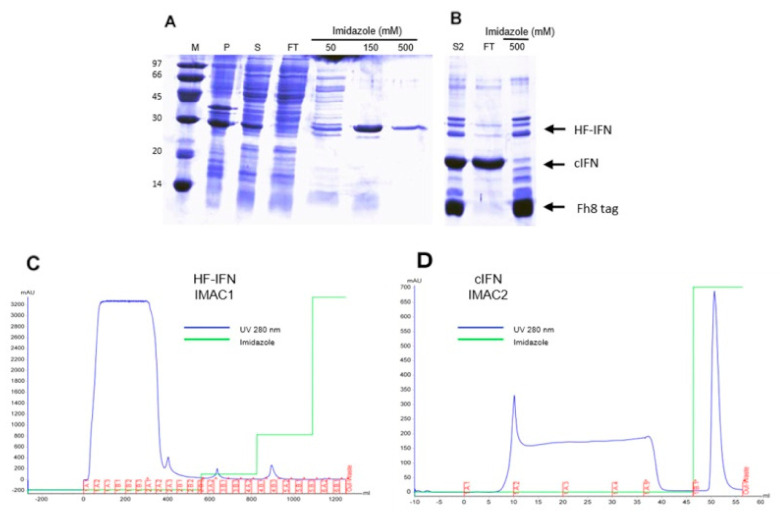
Purification of HF-IFN by IMAC-Ni^2+^ before and after tag removal. (**A**) and (**B**) SDS-PAGE of fractions collected from HF-IFN purification, before and after tag removal, respectively. (**C**) Chromatogram of HF-IFN purification before tag removal. (**D**) Chromatogram of cIFN purification after tag removal: untagged cIFN was recovered in flowthrough fraction, non-hydrolyzed protein, TEV protease and hydrolyzed solubility tag in eluted fraction (500 mM imidazole). (M) Molecular marker, (P) pellet after cell lysis (insoluble fraction), (S) soluble fraction, (FT) flowthrough. HF-IFN has 28.8kDa, and cIFN has a 19.4 kDa.

When HF-IFN was purified with the same IMAC/IMAC method as described above for HD-IFN, we were not able to recover pure untagged cIFN, as some of the contaminants remained in the sample (Figure 8B). Since the amount of cIFN recovered from HF-IFN after hydrolysis was much greater than that obtained after HD-IFN hydrolysis, we decided to improve the purification process of HF-IFN before the tag removal.

**Figure 9:**
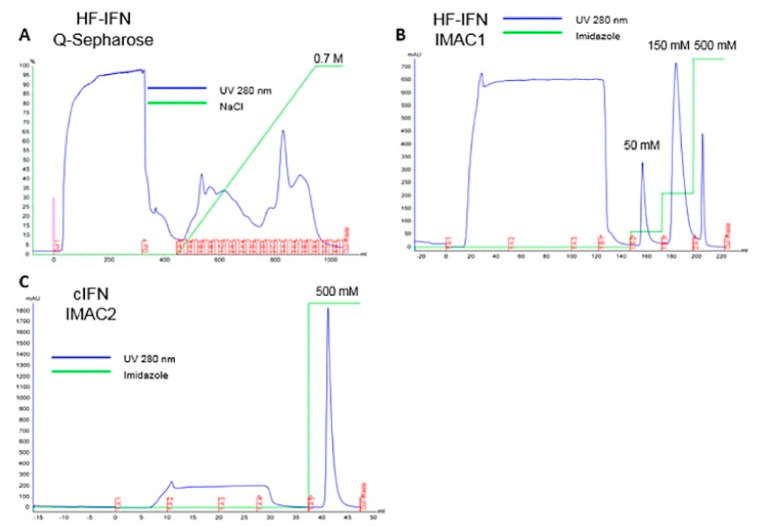
Chromatograms of HF-IFN and untagged cIFN purification process. (**A**) Q-Sepharose step, elution was performed using a linear gradient of 0 to 0.7 M NaCl. (**B**) IMAC1 step, HF-IFN was eluted with 150 mM imidazole. (**C**) IMAC2 step, after hydrolysis for tag removal. Untagged cIFN was recovered in flowthrough. All chromatographies were performed in ÄKTA Avant equipment.

In order to enhance cIFN purity, an additional Q-Sepharose purification step was added in the HF-IFN purification process prior to IMAC/IMAC. The chromatograms of HF-IFN and cIFN purification are displayed in Figure 9. After introducing Q-Sepharose step, we were able to recover a higher amount of HF-IFN in IMAC1. Hence, a higher UV 280 nm peak at 150 mM imidazole elution was observed in IMAC1 after Q-Sepharose when compared to 150 mM imidazole elution without Q-Sepharose (Figure 8C and Figure 9B).

**Figure 10:**
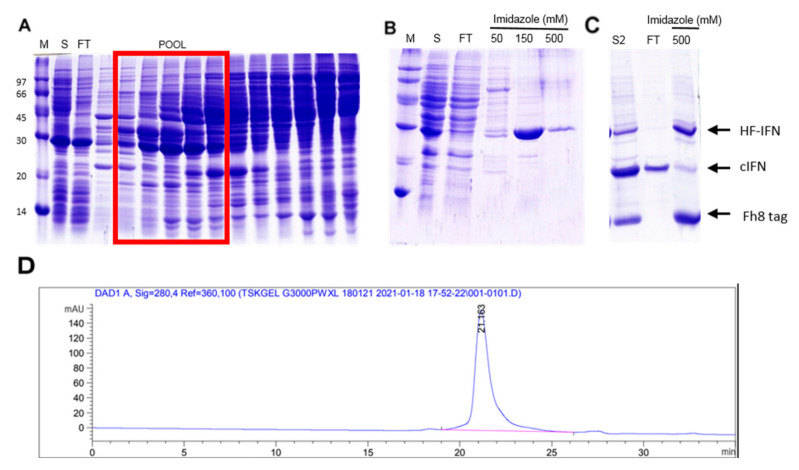
Purification of HF-IFN by Q-Sepharose and IMAC-Ni^2+^ before and after tag removal. (**A**) Q-Sepharose step. The red square represents the elution pool used for further purification steps. (**B**) IMAC1 step (**C**) IMAC2 step, after hydrolysis. Untagged cIFN was collected in flowthrough. (**D**) HPSEC chromatogram showing the retention time of cIFN peak (21.163 min); the peak integration displayed 100% purity of cIFN peak. (M) molecular marker, (S) soluble fraction, (FT) flowthrough. HF-IFN has 28.8 kDa and cIFN has a 19.4 kDa.

The HF-IFN pool was collected from Q-Sepharose (Figure 10A) and submitted to a subsequent IMAC step. HF-IFN was recovered after 150 mM elution (Figure 10B). The hydrolyzed cIFN from HF-IFN molecule was recovered in the flowthrough fraction of IMAC-Ni^2+^, while the non-hydrolyzed protein remained adsorbed to the chromatographic column as well as the hydrolyzed solubility tag (Figure 10C). While we were not able to recover pure cIFN in the previous experiments, the cIFN derived from HF-IFN submitted to this purification protocol was 100% pure when analyzed by HPSEC (Figure 10D).

H-, HD- and HF-IFN purification details are displayed in Table 2. The amount of soluble load was higher in the HD-IFN purification process than in HF-IFN. Nonetheless, the step recovery of the first chromatography step was much higher in HF-IFN purification than in HD-IFN (Table 2), confirming the results obtained when only IMAC was employed in the purification process (Figure 8). As explained above, the amount of H-IFN in the soluble load was minimal, leading to the lowest recovery in H-IFN process. The final recovery of soluble untagged cIFN after hydrolysis achieved 8 mg/L (58% recovery) and 1.44 mg/L (14% recovery), derived from HF-IFN and HD-IFN, respectively. The final yield of soluble untagged cIFN from HF-IFN construct was 5.5-fold higher when compared to HD-IFN (Table 3). Therefore, the viral infection assays were performed using cIFN derived from the HF-IFN construction.

### 3.6. cIFN Exerts Antiviral Activity in Experimental Model of Arboviruses and SARS-CoV-2 Infection in VERO Cells

**Figure 11:**
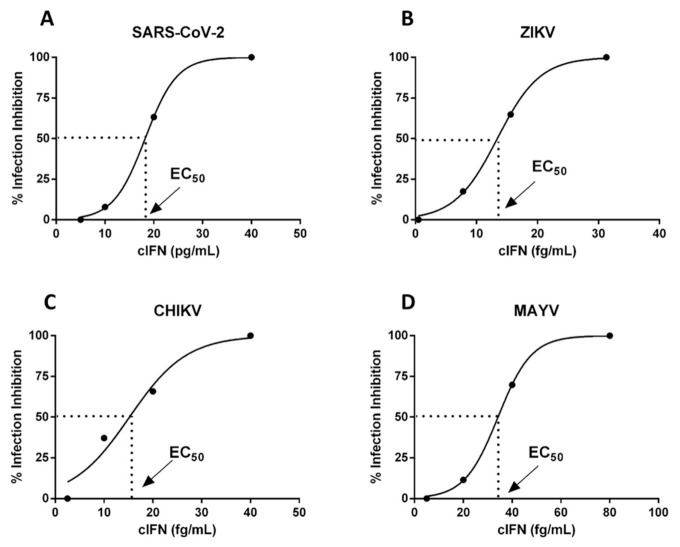
EC_50_ sigmoid graph of cIFN_._ Each graph represents one of the three replicates. Results were obtained from three independent experiments performed in triplicate and are displayed as (**A**) SARS-CoV-2, (**B**) Zika virus, (**C**) Chikungunya virus and (**D**) Mayaro virus. Values refer to half maximal effective concentration of cIFN which inhibits virus infection.

The activity of IFN consensus (HF-IFN-derived) was evaluated in infection experiments using Vero cells, Zika, Mayaro, Chikungunya and SARS-CoV-2 viruses. The EC_50_ values of cIFN were determined in three independent experiments for each virus using replicates. Very low concentrations of the molecule were sufficient to inhibit virus cytopathic effects, with EC_50_ of 35.7 ± 5.1 fg/mL for Mayaro, 12.1 ± 1.8 fg/mL for Chikungunya, 14.2 ± 8fg/mL for Zika and 11 ± 7 pg/mL for SARS-CoV-2 (Figure 11 and Table 4). In previous assays, the effectivity of interferon against Zika using this same test was determined as αIFN 2a EC_50_: 1.01 ng/mL, and αIFN 2b EC_50_: 0.02 ng/mL.

## 4. Discussion

The *E. coli* bacterium was the pioneer expression platform in the production of biopharmaceuticals and was consolidated in industrial manufacturing. *E. coli* is widely used due to its high growth speed, low complexity in genetic manipulations, and high yield in the synthesis of recombinant proteins (approximately 30–50% of total proteins) [45,46]. Currently, several drugs are produced in *E. coli*, such as: human insulin, interferon-α2a, anakinra, somatropin, among many others [47].

Among the existing variants of interferons, there is an artificial molecule called consensus. This variant has the characteristic of having the most prevalent amino acid sequence found in 14 subtypes of human interferons. Despite demonstrating higher biological activity than endogenous IFN-α, the production of cIFN remains a challenge [26]. Researchers were able to obtain cIFN in insoluble structures, the bacterial inclusion bodies. Thus, additional steps are needed to isolate, solubilize, purify and refold the molecule to obtain the biologically active cIFN [27,28]. We aimed to improve the solubility of cIFN produced in recombinant *E. coli*.

A common approach to overcome inclusion body formations is the use of solubility tags. This strategy may not only increase soluble protein recovery but may also aid in several purification methods [48,49]. Taking into account that 80% of the investments spent in this area are directly related to the recovery and purification of proteins, new solutions that simplify these processes, such as efficient solubility tags, are essential in biotechnological progress [15]. Although solubility tags may seem a great strategy to overcome inclusion body formation, it is known that there is no perfect tag. Thus, for obtaining soluble recombinant proteins in *E. coli*, different tags must be assessed individually as these molecules may behave differently according to the proprieties of the target protein [14]. 

A wide diversity of solubility tags is commercially available: maltose-binding protein (MBP), N-utilization substance (NusA), thioredoxin (TrxA), glutathione S-transferase (GST) and small ubiquitin-related modifier (SUMO) [50]. Even though these tags are currently used for research in bench-scale and also large-scale production, these fusion tags commonly display low yields of protein due to unspecific interactions with the respective matrices [51], and only SUMO was applied to cIFN production [26]. In 2012, Costa et al. compared the performance of Fh8 and the aforementioned tags using six difficult-to-express proteins. The Fh8 tag enhanced protein solubility as the commonly used Trx, NusA or MBP partners. Fh8 tag not only acts as an effective solubility enhancer, but also has the advantage of providing greater stability and allows easier evaluation of the target protein in the purification process, due to its low molecular weight [14].

The solubility tags used in this study were placed on the N-terminal portion of the target protein. The insertion of tags in the N-terminal portion is recommended, as it becomes the first portion to be translated. This strategy has the main objective to aid in the correct folding of the molecule of interest. In this way, tagged proteins in the N-terminal region were shown to have better folding when compared to cloning using tags in the C-terminal region [52]. Comparison of the three constructs of recombinant interferon aimed to evaluate the solubility of cIFN genetically fused to the tags Fh8 and DsbC (HF-IFN and HD-IFN, respectively), as well as in the absence of solubility tags and fused only with the affinity His-tag (H-IFN). We show that the production of cIFN in the absence of solubility tags (H-IFN) led to the aggregation and formation of inclusion bodies. When using DsbC or Fh8 tags, we were able to express soluble HD- and HF-IFN.

Our results are in agreement with the literature data that showed that Fh8 was able to increase solubility of several proteins, being as effective as the well-known MBP, NusA and Trx tags [15] and SUMO [53]. Expression of the *cifn* gene fused to the Fh8 tag (HF-IFN) not only promoted greater protein solubility, but also served to maintain the protein in the soluble fraction for a longer time during *E. coli* cultivation than the protein without solubility tag (H-IFN), which aggregated into bacterial inclusion bodies. These findings suggest that Fh8 could be a proper fusion tag for scaling up the bioprocess, since the downstream processing is also facilitated when there is less tendency of protein precipitation. To date, there are no reports in the literature relating recombinant cIFN synthesis associated with Fh8 in the *E. coli* expression platform.

DsbC is another solubility tag that was reported as being especially effective for proteins with disulfide bonds [17,54]. Here, the use of the DsbC tag also augmented the solubility of cIFN, which has two disulfide bonds, but it was less efficient than the Fh8 tag. Notwithstanding, there is no universal rule for choosing the most effective solubility tag for every protein produced in the *E. coli* platform. It is suggested that each protein should be individually tested with different tags for better performance evaluation [14].

The size of the tag must be carefully considered as a sizable tag, comprising a wide range of amino acids, may cause metabolic overburden and not promote protein solubility [55]. DsbC is a large tag with 23.4 kDa, while Fh8 is much smaller, with only 7.6 kDa. This may explain the lower yield of HD-IFN (DsbC tag) than HF-IFN (Fh8 tag) in *E. coli* cultures. Furthermore, while DsbC represents 52% of HD-IFN molecule, Fh8 represents only 28% of HF-IFN. Thus, the production of 1 g of HF-IFN represents 0.72 g cIFN, while 1.0 g of HD-IFN contains 0.48 g of cIFN. SUMO has an intermediate size, 11.5 kDa, and represents 36% of SUMO-cIFN, as previously reported [26].

Besides the use of solubility tags, it is also known that higher temperatures favor rapid bacterial growth, resulting in a higher production rate of the target protein and possibly in protein aggregation and the formation of inclusion bodies. Furthermore, the presence of strong promoters, such as the T7 promoter, high concentrations of IPTG, absence of chaperones and hydrophobic regions of proteins are complementary factors for the formation of inclusion bodies [56,57].

The decrease of the culture temperature from 37 °C to 30 °C as well as the concentration of IPTG in the culture medium from 1 mM to 0.1 mM favored HF-IFN production in the soluble fraction. These results are in agreement with literature data that reported an increase of recombinant IFN-α solubility by decreasing the temperature and reducing the IPTG concentration [58]. This finding can be explained by the fact that lower concentrations of IPTG favor slower protein translation, which allows correct protein folding. In addition, lower temperatures during the culture induction phase (16 to 25 °C) may result in an increase of the final yield of the protein in the soluble form [59], a phenomenon also related to slower protein synthesis [60,61,62,63,64,65].

The synthesis of HF-IFN and HD-IFN was also evaluated in three culture media: autoinduction, HDF and SDAB. These three media are suitable for good manufacturing practices (GMP), while LB medium is not, due to tryptone presence [66], and it does not contain a carbon source, leading to low biomass production as shown before. In addition, Erlenmeyer flasks were replaced by TunAir flasks, because they allow higher oxygen transfer rates due to their geometry and the air filter that permits superior gas exchange. These characteristics of TunAir flasks approach them to the bioreactor conditions [67]. Among the conditions and constructs tested, the Fh8-fused variant (HF-IFN) produced in an autoinduction medium at 30 °C was the most efficient for increasing the protein solubility (Figure 4 and Figure 5). The production of DsbC-fused variant (HD-IFN) in the same medium and temperature yielded lower amount of soluble protein than HF-IFN (Figure 6 and Figure 7). Comparing the maximum production points of HF-IFN or HD-IFN in each culture medium (Figure S9), the production of HF-IFN in the autoinduction medium at 30 °C is the best for a larger production of cIFN. El-Baky et al. reports increased protein concentration in the soluble fraction when the culture temperature decreased from 37 °C to 25 °C. Besides, the author showed that the production of cIFN in autoinduction medium was 1.8 times higher (270 mg/L) when compared to an IPTG-mediated induction system (150 mg/L) [68]. 

The autoinduction culture medium [41] supplies amino acids, vitamins and protein hydrolysates for bacterial growth. These elements support the demand for bacterial metabolism and provide high values of specific growth rate, μ [69]. In addition, lactose induction is more convenient as there is no need to monitor growth to add the inducer; it also provides higher cell density and target protein concentration when compared to IPTG-induced media [41]. Lactose can also serve as a source of carbon and energy, which contributes to increased biomass at the end of cultivation. In contrast, IPTG induction may generate a deficiency in nutrient uptake, impacting on bacterial growth and production of the target protein [68]. Hence, the autoinduction culture medium has superior commercial viability in large-scale production as this medium promotes high cell density cultures in batch cultures [70]. This would simplify bioprocess implementation on an industrial scale in comparison to fed-batch strategies [70].

Even though there is no massive data about cIFN purification from *E. coli*, recombinant IFN-α is also produced as inclusion bodies, refolded, and recovered. Many authors related their experience in purifying IFN-α from inclusion bodies mainly using Q-Sepharose [71,72,73] and DEAE Sepharose [28,74]. While we were not able to recover pure cIFN from HD-IFN molecule, the untagged cIFN obtained from HF-IFN showed 100% purity by HPSEC after TEV hydrolysis, with a yield of 8 mg/L. Soluble cIFN production was reported by El-Baky et al. [68] when produced in autoinduction medium using *E. coli* BL21-CodonPlus(DE3), which is a lineage of *E. coli* different from that used in this work. The author reports a recovery yield of 270 mg/L with a single-step DEAE-Sepharose chromatography. This result seems promising; however, we were not able to achieve the same results as reported by those authors without a solubility tag. In addition, Peciak et al. expressed soluble cIFN fused with solubility SUMO tag [26] with a yield of 50 mg/L. SUMO-cIFN expression was carried in *E. coli SHuffle*^®^ suggesting that *E. coli* lineage may be crucial in order to obtain soluble cIFN. We also highlight the need for several chromatographic steps to exclude and discard contaminants that are together with cIFN in this process. These results indicate a favorable bioprocess for cIFN production as soluble protein, yet the production and purification processes must be polished.

The functional characteristics of IFN-α are widely studied; however, the use of cIFN, a non-natural synthetic recombinant interferon, has not been described in the context of ZIKV, MAYV, CHIKV and SARS-CoV-2 virus until now [25,75]. Our results indicate that the free cIFN obtained after tag removal exerted strong antiviral activity against all these viruses. When cIFN was added to VERO cells before virus infection, cultures showed a lower viral-induced cytopathic effect, showing that the treatment with cIFN previously to the viral infection was able to inhibit viral multiplication. As an example of the strong activity of cIFN, the non-treated control showed almost complete destruction of the cell monolayer in 24 h of MAYV infection, whereas cells treated with only 81.4 fg/mL and 162.8 fg/mL showed around 80% and 100% confluency, respectively. Hence, our results reiterate a possible therapeutic utilization of cIFN in the control of the described arboviruses infection.

## 5. Conclusions

The production of cIFN fused to the Fh8 tag (HF-IFN) favored protein solubility when compared to the H- and HD-IFN fusion molecules. Furthermore, *E. coli* BL21(DE3) cultivation for production of HF-IFN in autoinduction medium at 30 °C presented a final recovery of soluble cIFN of 8 mg/L, 5.5-fold higher when compared to HD-IFN. Thus, the production of cIFN fused to the Fh8 tag, in an autoinduction medium at 30 °C, is proposed as a viable approach to obtain this molecule in the soluble fraction. Even though we recovered low amounts of target protein, the aim to produce soluble cIFN could be an excellent alternative to inclusion bodies and should be further explored.

Most important, our results demonstrate, for the first time, that the cIFN produced was able to inhibit the cytopathic effect of the ZIKV, MAYV, CHIKV and SARS-CoV-2 virus in VERO cells. Of note, this is a class of molecules known and approved by regulatory agencies such as the Food and Drug Administration (FDA), a fact that would accelerate a possible insertion of the molecule in a clinical setting in cases of public health emergencies, when the risks associated with IFN side-effects compensate the risks related to the disease [76,77].

## Figures and Tables

**Table 1:** Constructs for production of recombinant cIFN.

Protein	TAG	Gene Size	Protein Size
H-IFN	6xHis	522 bp	20.2 kDa
HF-IFN	6xHis-Fh8	753 bp	28.8 kDa
HD-IFN	6xHis-DsbC	1194 bp	44.6 kDa

**Table 2:** Comparison of purification steps of HF-, HD- and H-IFN. #H-IFN concentration in soluble load was not sufficient for detection and the detected H-IFN in IMAC elution (4.74 mg) was considered the amount in the soluble load.

**Fraction**	**Total Protein (mg)**	**Relative Purity (%)**	**HF-IFN (mg)**	**Step Recovery (%)**	**Total Recovery (%)**	**Purification Factor**
**Soluble Load**	456	15.4	70.5	100	100	-
**Q-Sepharose FF Elution**	112	17.4	19.5	27.8	27.8	1.12
**IMAC Elution**	5	81.6	4.08	21	5.8	4.69
**Fraction**	**Total Protein (mg)**	**Relative Purity (%)**	**HD-IFN (mg)**	**Step Recovery (%)**	**Total Recovery (%)**	**Purification Factor**
**Soluble Load**	2649	52.7	1397.3	100	100	-
**IMAC Elution**	68	32.7	22.2	1.6	1.6	0.62
**Fraction**	**Total Protein (mg)**	**Relative Purity (%)**	**H-IFN (mg)**	**Step Recovery (%)**	**Total Recovery (%)**	
**Pellet**	835	58	484.8			
**Soluble Load**	832	0	4.74#	100	100	
**IMAC Elution**	52	9	4.74	100	1	

**Table 3:** Comparison of untagged cIFN recovery after hydrolysis for tag removal.

Construct	Untagged cIFN Recovery after Hydrolysis (%)
**HF-IFN -> cIFN**	58
**HD-IFN -> cIFN**	14

**Table 4:** EC50 values for virus inhibition with the use of cIFN.

cIFN + Virus	EC_50_
**Zika (ZIKV)**	14 ± 8 fg/mL
**Mayaro (MAYV)**	35 ± 5 fg/mL
**Chikungunya (CHIV)**	12 ± 2 fg/mL
**SARS-CoV-2**	11 ± 7 fg/mL

## Data Availability

All data is contained within this article or supplementary materials.

## Supplementary Materials

The following are available online at https://www.mdpi.com/article/10.3390/life11060460/s1, Figure S1a: Nucleotide sequence of the HF-IFN construct and site for TEV hydrolysis in the pET28a vector, Figure S1b: Nucleotide sequence of the HD-IFN construct and site for TEV hydrolysis in pET28a vector, Figure S1c: Nucleotide sequence of the H-IFN construct in pET28a vector, Figure S2: Restriction digest analysis for confirmation of the presence of the insert in the commercial plasmid, Figure S3: Preparative electrophoresis for HF-IFN insert and pET28a expression vector purification, Figure S4: DsbC and pET28a cloning steps, Figure S5: Selection of positive colonies for the HF-IFN insert, Figure S6: Positive *E. coli* colonies for plasmid pET28a-HD-IFN, Figure S7: Electrophoresis gel for PCR-amplified H-IFN insert detection and selection of bacterial colonies, Figure S8: Quantification analysis of H- and HF-IFN in the soluble fraction, Figure S9. Comparison of HF-IFN and HD-IFN maximum production in different culture media and temperatures, Figure S10. Enzymatic kinetics of HF-IFN hydrolysis in the presence of TEV, Table S1: Sequence of nucleotides used in primer design, Table S2a: Composition of LB-agar medium, Table S2b: Composition of the complex medium of autoinduction, Table S2c: Composition of the chemically defined medium SDAB, Table S2d: Composition of chemically defined medium HDF.

## References

[1] RNCOS Global Protein Therapeutics Market Outlook 2020. https://www.researchandmarkets.com/reports/3422491/global-protein-therapeutics-market-outlook-2020.

[2] Kesik-Brodacka M. (2018). Progress in biopharmaceutical development. Biotechnol. Appl. Biochem..

[3] Huang X., Wang X., Zhang J., Xia N., Zhao Q. (2017). *Escherichia coli*-derived virus-like particles in vaccine development. npj Vaccines.

[4] Kesik-Brodacka M., Romanik A., Mikiewicz-Sygula D., Plucienniczak G., Plucienniczak A. (2012). A novel system for stable, high-level expression from the T7 promoter. Microb. Cell Fact..

[5] Terpe K. (2006). Overview of bacterial expression systems for heterologous protein production: From molecular and biochemical fundamentals to commercial systems. Appl. Microbiol. Biotechnol..

[6] Demain A.L., Vaishnav P. (2009). Production of recombinant proteins by microbes and higher organisms. Biotechnol. Adv..

[7] Pacheco B., Crombet L., Loppnau P., Cossar D. (2012). A screening strategy for heterologous protein expression in *Escherichia coli* with the highest return of investment. Protein Expr. Purif..

[8] Correa A., Oppezzo P. (2014). Overcoming the solubility problem in *E. coli*: Available approaches for recombinant protein production. Insoluble Proteins: Methods and Protocols.

[9] Hammarstrom M., Hellgren N., van Den Berg S., Berglund H., Hard T. (2002). Rapid screening for improved solubility of small human proteins produced as fusion proteins in *Escherichia coli*. Protein Sci..

[10] Esposito D., Chatterjee D.K. (2006). Enhancement of soluble protein expression through the use of fusion tags. Curr. Opin. Biotechnol..

[11] Vincentelli R., Cimino A., Geerlof A., Kubo A., Satou Y., Cambillau C. (2011). High-throughput protein expression screening and purification in *Escherichia coli*. Methods.

[12] Nozach H., Fruchart-Gaillard C., Fenaille F., Beau F., Ramos O.H.P., Douzi B., Saez N.J., Moutiez M., Servent D., Gondry M. (2013). High throughput screening identifies disulfide isomerase DsbC as a very efficient partner for recombinant expression of small disulfide-rich proteins in *E. coli*. Microb. Cell Factories.

[13] Silva E., Castro A., Lopes A., Rodrigues A., Dias C., Conceição A., Alonso J., Correia da Costa J.M., Bastos M., Parra F. (2004). A recombinant antigen recognized by Fasciola hepatica-infected hosts. J. Parasitol..

[14] Costa S.J., Almeida A., Castro A., Domingues L., Besir H. (2013). The novel Fh8 and H fusion partners for soluble protein expression in *Escherichia coli*: A comparison with the traditional gene fusion technology. Appl. Microbiol. Biotechnol..

[15] Costa S.J., Coelho E., Franco L., Almeida A., Castro A., Domingues L. (2013). The Fh8 tag: A fusion partner for simple and cost-effective protein purification in *Escherichia coli*. Protein Expr. Purif..

[16] Conceição M., Costa S., Castro A., Almeida A. (2010). Fusion Proteins, Its Preparation Process and Its Application on Recombinant Protein Expression Systems. Portugal Patent.

[17] Zhuo X.-F., Zhang Y.-Y., Guan Y.-X., Yao S.-J. (2014). Co-expression of disulfide oxidoreductases DsbA/DsbC markedly enhanced soluble and functional expression of reteplase in *Escherichia coli*. J. Biotechnol..

[18] Baneyx F., Mujacic M. (2004). Recombinant protein folding and misfolding in *Escherichia coli*. Nat. Biotechnol..

[19] Zapun A., Creighton T.E., Missiakas D., Raina S. (1995). Structural and Functional Characterization of DsbC, a Protein Involved in Disulfide Bond Formation in *Escherichia coli*. Biochemistry.

[20] Sun X.X., Wang C.C. (2000). The N-terminal sequence (residues 1–65) is essential for dimerization, activities, and peptide binding of *Escherichia coli* DsbC. J. Biol. Chem..

[21] Turchetto J., Sequeira A.F., Ramond L., Peysson F., Brás J.L., Saez N.J., Duhoo Y., Blémont M., Guerreiro C.I., Quinton L. (2017). High-throughput expression of animal venom toxins in *Escherichia coli* to generate a large library of oxidized disulphide-reticulated peptides for drug discovery. Microb. Cell Factories.

[22] Joly J.C., Leung W.S., Swartz J.R. (1998). Overexpression of *Escherichia coli* oxidoreductases increases recombinant insulin-like growth factor-I accumulation. Proc. Natl. Acad. Sci. USA.

[23] Fish E.N., Harrison S.A., Hassanein T. (2008). The role of consensus interferon in the current treatment of chronic hepatitis C viral infection. Gastroenterol. Hepatol..

[24] Ozes O.N., Reiter Z., Klein S., Blatt L.M., Taylor M.W. (1992). A comparison of interferon-Con1 with natural recombinant interferons-alpha: Antiviral, antiproliferative, and natural killer-inducing activities. J. Interferon Res..

[25] Blatt L.M., Davis J.M., Klein S.B., Taylor M.W. (1996). The biologic activity and molecular characterization of a novel synthetic interferon-alpha species, consensus interferon. J. Interferon Cytokine Res..

[26] Peciak K., Tommasi R., Choi J.W., Brocchini S., Laurine E. (2014). Expression of soluble and active interferon consensus in SUMO fusion expression system in *E. coli*. Protein Expr. Purif..

[27] Wang F., Liu Y., Li J., Ma G., Su Z. (2006). On-column refolding of consensus interferon at high concentration with guanidine-hydrochloride and polyethylene glycol gradients. J. Chromatogr. A.

[28] Mohammed Y., El-Baky N.A., Redwan E.M. (2012). Expression, purification, and characterization of recombinant human consensus interferon-alpha in *Escherichia coli* under λP(L) promoter. Prep. Biochem. Biotechnol..

[29] Rodriguez A.K., Muñoz A.L., Segura N.A., Rangel H.R., Bello F. (2019). Molecular characteristics and replication mechanism of dengue, zika and chikungunya arboviruses, and their treatments with natural extracts from plants: An updated review. EXCLI J..

[30] Vairo F., Haider N., Kock R., Ntoumi F., Ippolito G., Zumla A. (2019). Chikungunya: Epidemiology, Pathogenesis, Clinical Features, Management, and Prevention. Infect. Dis. Clin. N. Am..

[31] Acosta-Ampudia Y., Monsalve D.M., Rodríguez Y., Pacheco Y., Anaya J.M., Ramírez-Santana C. (2018). Mayaro: An emerging viral threat?. Emerg. Microbes Infect..

[32] Colón-González F.J., Peres C.A., Steiner São Bernardo C., Hunter P.R., Lake I.R. (2017). After the epidemic: Zika virus projections for Latin America and the Caribbean. PLoS Negl. Trop. Dis..

[33] Goebel S., Snyder B., Sellati T., Saeed M., Ptak R., Murray M., Bostwick R., Rayner J., Koide F., Kalkeri R. (2016). A sensitive virus yield assay for evaluation of Antivirals against Zika Virus. J. Virol. Methods.

[34] Gorbalenya A.E., Baker S.C., Baric R.S., de Groot R.J., Drosten C., Gulyaeva A.A., Haagmans B.L., Lauber C., Leontovich A.M., Neuman B.W. (2020). The species Severe acute respiratory syndrome-related coronavirus: Classifying 2019-nCoV and naming it SARS-CoV-2. Nat. Microbiol..

[35] Harrison A.G., Lin T., Wang P. (2020). Mechanisms of SARS-CoV-2 Transmission and Pathogenesis. Trends Immunol..

[36] World Health Organization (2021). WHO Coronavirus Disease (COVID-19) Dashboard.

[37] Hu B., Guo H., Zhou P., Shi Z.-L. (2020). Characteristics of SARS-CoV-2 and COVID-19. Nat. Rev. Microbiol..

[38] Awadasseid A., Wu Y., Tanaka Y., Zhang W. (2021). Current advances in the development of SARS-CoV-2 vaccines. Int. J. Biol. Sci..

[39] Bouayad A. (2020). Innate immune evasion by SARS-CoV-2: Comparison with SARS-CoV. Rev. Med. Virol..

[40] De Spiegeleer P., Sermon J., Lietaert A., Aertsen A., Michiels C.W. (2004). Source of tryptone in growth medium affects oxidative stress resistance in *Escherichia coli*. J. Appl. Microbiol..

[41] Studier F.W. (2005). Protein production by auto-induction in high-density shaking cultures. Protein Expr. Purif..

[42] Campani G., dos Santos M.P., da Silva G.G., Horta A.C.L., Badino A.C., de Campos Giordano R., Gonçalves V.M., Zangirolami T.C. (2016). Recombinant protein production by engineered *Escherichia coli* in a pressurized airlift bioreactor: A techno-economic analysis. Chem. Eng. Process. Process Intensif..

[43] Li Z., Kessler W., Van Den Heuvel J., Rinas U. (2011). Simple defined autoinduction medium for high-level recombinant protein production using T7-based *Escherichia coli* expression systems. Appl. Microbiol. Biotechnol..

[44] Seeger A., Schneppe B., McCarthy J.E.G., Deckwer W.D., Rinas U. (1995). Comparison of temperature- and isopropyl-beta-d-thiogalacto-pyranoside-induced synthesis of basic fibroblast growth factor in high-cell-density cultures of recombinant *Escherichia coli*. Enzym. Microb. Technol..

[45] Sahdev S., Khattar S.K., Saini K.S. (2007). Production of active eukaryotic proteins through bacterial expression systems: A review of the existing biotechnology strategies. Mol. Cell. Biochem..

[46] Rodríguez V., Asenjo J.A., Andrews B.A. (2014). Design and implementation of a high yield production system for recombinant expression of peptides. Microb. Cell Fact..

[47] Baeshen M.N., Al-Hejin A.M., Bora R.S., Ahmed M.M.M., Ramadan H.A.I., Saini K.S., Baeshen N.A., Redwan E.M. (2015). Production of Biopharmaceuticals in *E. coli*: Current Scenario and Future Perspectives. J. Microbiol. Biotechnol..

[48] Mahmoudi Gomari M., Saraygord-Afshari N., Farsimadan M., Rostami N., Aghamiri S., Farajollahi M.M. (2020). Opportunities and challenges of the tag-assisted protein purification techniques: Applications in the pharmaceutical industry. Biotechnol. Adv..

[49] Protection against Recurrent Genital Herpes by Therapeutic Immunization with Herpes Simplex Virus Type 2 Ribonucleotide Reductas BenMohamed. https://uspto.report/patent/app/20200046827.

[50] Costa S., Almeida A., Castro A., Domingues L. (2014). Fusion tags for protein solubility, purification, and immunogenicity in *Escherichia coli*: The novel Fh8 system. Front. Microbiol..

[51] Arnau J., Lauritzen C., Petersen G.E., Pedersen J. (2006). Current strategies for the use of affinity tags and tag removal for the purification of recombinant proteins. Protein Expr. Purif..

[52] Dyson M.R., Shadbolt S.P., Vincent K.J., Perera R.L., McCafferty J. (2004). Production of soluble mammalian proteins in *Escherichia coli*: Identification of protein features that correlate with successful expression. BMC Biotechnol..

[53] Zhang M., Wang Z., Chi L., Sun J., Shen Y. (2018). Enhanced production of soluble tumor necrosis factor-related apoptosis-inducing ligand in *Escherichia coli* using a novel self-cleavable tag system Fh8-ΔI-CM. Protein Expr. Purif..

[54] Zhang Z., Li Z.-H., Wang F., Fang M., Yin C.-C., Zhou Z.-Y., Lin Q., Huang H.-L. (2002). Overexpression of DsbC and DsbG markedly improves soluble and functional expression of single-chain Fv antibodies in *Escherichia coli*. Protein Expr. Purif..

[55] Malhotra A. (2009). Tagging for Protein Expression. Methods Enzymol..

[56] Singh S.M., Panda A.K. (2005). Solubilization and refolding of bacterial inclusion body proteins. J. Biosci. Bioeng..

[57] Carrió M.M., Cubarsi R., Villaverde A. (2000). Fine architecture of bacterial inclusion bodies. FEBS Lett..

[58] Rabhi-Essafi I., Sadok A., Khalaf N., Fathallah D.M. (2007). A strategy for high-level expression of soluble and functional human interferon alpha as a GST-fusion protein in *E. coli*. Protein Eng. Des. Sel..

[59] Vera A., González-Montalbán N., Arís A., Villaverde A. (2007). The conformational quality of insoluble recombinant proteins is enhanced at low growth temperatures. Biotechnol. Bioeng..

[60] Baldwin R.L., Baldwin R.L. (1986). Temperature Dependence of the Hydrophobic Interaction in Protein Folding Temperature dependence of the hydrophobic interaction in protein folding (hydrocarbon model). Proc. Natl. Acad. Sci. USA.

[61] Betts S.D., King J. (1998). Cold rescue of the thermolabile tailspike intermediate at the junction between productive folding and off-pathway aggregation. Protein Sci..

[62] Pope W.H., Haase-Pettingell C., King J. (2004). Protein folding failure sets high-temperature limit on growth of phage P22 in Salmonella enterica serovar Typhimurium. Appl. Environ. Microbiol..

[63] Scharnagl C., Reif M., Friedrich J. (2005). Stability of proteins: Temperature, pressure and the role of the solvent. Biochim. Biophys. Acta-Proteins Proteom..

[64] Schellman J.A. (1997). Temperature, stability, and the hydrophobic interaction. Biophys. J..

[65] Strandberg L., Enfors S.O. (1991). Factors influencing inclusion body formation in the production of a fused protein in *Escherichia coli*. Appl. Environ. Microbiol..

[66] World Health Organization (2003). WHO Guidelines on Transmissible Spongiform Encephalopathies in Relation to Biological and Pharmaceutical Products.

[67] Takahashi M., Aoyagi H. (2018). Practices of shake-flask culture and advances in monitoring CO_2_ and O_2_. Appl. Microbiol. Biotechnol..

[68] EL-Baky N.A., Linjawi M.H., Redwan E.M. (2015). Auto-induction expression of human consensus interferon-alpha in *Escherichia coli*. BMC Biotechnol..

[69] Brown T. (2003). Clonagem Gênica e Análise de DNA-Uma Introdução.

[70] Cardoso V.M., Campani G., Santos M.P., Silva G.G., Pires M.C., Gonçalves V.M., Giordano R.D.C., Sargo C.R., Horta A.C., Zangirolami T.C. (2020). Cost analysis based on bioreactor cultivation conditions: Production of a soluble recombinant protein using *Escherichia coli* BL21(DE3). Biotechnol. Rep..

[71] Meng J., Yan Z., Xue X., Hao Q., Wan Y., Qin X., Zhang C., You Y., Han W., Zhang Y. (2007). High-yield expression, purification and characterization of tumor-targeted IFN-α2a. Cytotherapy.

[72] Srivastava P., Bhattacharaya P., Pandey G., Mukherjee K.J. (2005). Overexpression and purification of recombinant human interferon alpha2b in *Escherichia coli*. Protein Expr. Purif..

[73] Babu K.R., Swaminathan S., Marten S., Khanna N., Rinas U. (2000). Production of interferon-α in high cell density cultures of recombinant *Escherichia coli* and its single step purification from refolded inclusion body proteins. Appl. Microbiol. Biotechnol..

[74] Ahmed N., Bashir H., Zafar A.U., Khan M.A., Tahir S., Khan F., Khan M.I., Akram M., Husnain T. (2015). Optimization of conditions for high-level expression and purification of human recombinant consensus interferon (rh-cIFN) and its characterization. Biotechnol. Appl. Biochem..

[75] El-Baky N.A., Redwan E.M. (2015). Therapeutic Alpha-Interferons Protein: Structure, Production, and Biosimilar. Prep. Biochem. Biotechnol..

[76] Hiratsuka M., Minakami H., Koshizuka S., Sato I. (2000). Administration of interferon-alpha during pregnancy: Effects on fetus. J. Perinat. Med..

[77] Egberts F., Lischner S., Russo P., Kampen W.U., Hauschild A. (2006). Diagnostic and therapeutic procedures for management of melanoma during pregnancy: Risks for the fetus?. J. Dtsch. Dermatol. Ges..

