# Supplementary material for: Strategies for the Production of Soluble Interferon-Alpha Consensus and Potential Application in Arboviruses and SARS-CoV-2
Source: Life (Basel). 2021 May 21;11(6):460. doi: 10.3390/life11060460 (PMC8223780; doi:10.3390/life11060460)
Supplement: Supplementary file 1 [file life-11-00460-s001.zip › life-1195215-supplementary.pdf]

Strategies for the production of soluble interferon-alpha *consensus* and potential application in Arboviruses and SARS-CoV-2

Felipe Grabarz [1,2], Alexandre Paulo Yague Lopes [1], Flávia Ferreira Barbosa [2,3], Giovana Cappio Barazzone [1], Jademilson C. Santos [1], Viviane Fongaro Botosso [4], Soraia Attie Calil Jorge [5], Ana Lucia Tabet Oller Nascimento [1], Renato Mancini Astray [3] and Viviane Maimoni Gonçalves [1,*]

[1] Laboratório de Desenvolvimento de Vacinas, Instituto Butantan. São Paulo-SP, 05503-900 Brasil.
[2] Programa Interunidades em Biotecnologia, Universidade de São Paulo. São Paulo-SP, Brasil.
[3] Laboratório de Imunologia Viral, Instituto Butantan. São Paulo-SP, 05503-900 Brasil.
[4] Laboratório de Virologia, Instituto Butantan. São Paulo-SP, 05503-900 Brasil.
[5] Laboratório de Biotecnologia Viral, Instituto Butantan. São Paulo-SP, 05503-900 Brasil.
[#] Viviane Maimoni Gonçalves. viviane.goncalves@butantan.gov.br

Address correspondence to Viviane Maimoni Gonçalves, Laboratory of Vaccine Development, Instituto Butantan, Av Vital Brasil, 1500, 05503-900, São Paulo, SP, Brazil. Phone: +55-11-2627-9821; E-mail: viviane.goncalves@butantan.gov.br

C A T C A T C A C C A C C A C C A T A T G C C G A G C G T T C A A G A A G T T G A A A
A A C T G C T G C A T G T T C T G G A T C G T A A T G G T G A T G G T A A A G T T A G
C G C A G A A G A A C T G A A A G C A T T T G C C G A T G A T A G C A A A T G T C C
G C T G G A T A G C A A T A A A A T C A A G G C C T T T A T C A A A G A G C A C G A
T A A A A A C A A A G A T G G C A A G C T G G A T C T G A A A G A A C T G G T T A G
C A T T C T G A G C A G C G A A A A C C T G T A T T T T C A G G G A T C C T G T G A T
C T G C C G C A G A C A C A T A G C C T G G G T A A T C G T C G T G C A C T G A T T C
T G C T G G C A C A G A T G C G T C G T A T T A G C C C G T T T A G C T G T C T G A A
A G A T C G T C A T G A T T T T G G T T T T C C G C A A G A G G A A T T T G A T G G C
A A C C A G T T T C A G A A A G C A C A G G C A A T T A G C G T T C T G C A T G A A
A T G A T T C A G C A G A C C T T T A A C C T G T T C A G C A C C A A A G A T A G C A
G C G C A G C A T G G G A T G A A A G C C T G C T G G A A A A A T T C T A T A C C G
A A C T G T A T C A G C A G C T G A A T G A T C T G G A A G C A T G T G T T A T T C A
A G A G G T T G G T G T T G A A G A A A C A C C G C T G A T G A A T G T T G A T A G
T A T T C T G G C C G T G A A A A A A T A C T T T C A G C G C A T T A C C C T G T A T
C T G A C C G A A A A A A A G T A T A G C C C G T G T G C A T G G G A A G T T G T T
C G T G C A G A A A T T A T G C G T A G C T T T A G C A T T A G C A C C A A T C T G C
A A G A A C G T C T G C G T C G C A A A G A A T A A T A A

Figure S1. a: Nucleotide sequence of the HF-IFN construct and site for TEV hydrolysis in the pET28a vector. Grey: His-tag; Blue: Fh8 tag; Orange: TEV hydrolysis site; Red: cIFN sequence and Green: STOP codon. The underlined sequence refers to the Bam *HI* restriction site.

CATCATCACCACCACCATGATGACGCGGCAATTCAACAAACGT
TAGCCAAAATGGGCATCAAAAGCAGCGATATTCAGCCCGCGC
CTGTAGCTGGCATGAAGACAGTTCTGACTAACAGCGGCGTGT
TGTACATCACCGATGATGGTAAACATATCATTCAGGGGCCAAT
GTATGACGTTAGTGGCACGGCTCCGGTCAATGTCACCAATAA
GATGCTGTTAAAGCAGTTGAATGCGCTTGAAAAAGAGATGAT
CGTTTATAAAGCGCCGCAGGAAAAACACGTCATCACCGTGTTT
ACTGATATTACCTGTGGTTACTGCCACAAACTGCATGAGCAAA
TGGCAGACTACAACGCGCTGGGGATCACCGTGCGTTATCTTGC
TTTCCCGCGCCAGGGGCTGGACAGCGATGCAGAGAAAGAAAT
GAAAGCTATCTGGTGTGCGAAAGATAAAAACAAAGCGTTTGA
TGATGTGATGGCAGGTAAAAGCGTCGCACCAGCCAGTTGCGA
CGTGGATATTGCCGACCATTACGCACTTGGCGTCCAGCTTGGC
GTTAGCGGTACTCCGGCAGTTGTGCTGAGCAATGGCACACTT
GTTCCGGGTTACCAGCCGCCGAAAGAGATGAAAGAATTTCTC
GACGAACACCAAAAAAATGACCAGCGGTAAAGAAAACCTGTAT
TTTCAG<u>GGATCC</u>TGTGATCTGCCGCAGACACATAGCCTGGGTA
ATCGTCGTGCACTGATTCTGCTGGCACAGATGCGTCGTATTAG
CCCGTTTAGCTGTCTGAAAGATCGTCATGATTTTGGTTTTCCG
CAAGAGGAATTTGATGGCAACCAGTTTCAGAAAGCACAGGCA
ATTAGCGTTCTGCATGAAATGATTCAGCAGACCTTTAACCTGT
TCAGCACCAAAGATAGCAGCGCAGCATGGGATGAAAGCCTGC
TGGAAAAATTCTATACCGAACTGTATCAGCAGCTGAATGATCT
GGAAGCATGTGTTATTCAAGAGGTTGGTGTTGAAGAAACACC
GCTGATGAATGTTGATAGTATTCTGGCCGTGAAAAAATACTTT
CAGCGCATTACCCTGTATCTGACCGAAAAAAAGTATAGCCCGT
GTGCATGGGAAGTTGTTCGTGCAGAAATTATGCGTAGCTTTA
GCATTAGCACCAATCTGCAAGAACGTCTGCGTCGCAAAGAAT
AATAA

Figure S1b: Nucleotide sequence of the HD-IFN construct and site for TEV hydrolysis in pET28a vector. Grey: His-tag; Blue: DsbC tag; Orange: TEV hydrolysis site; Red: cIFN sequence and Green: STOP codon. The underlined sequence refers to the Bam *HI* restriction site.

CATCATCACCACCACCATTGTGATCTGCCGCAGACACATAGCC
TGGGTAATCGTCGTGCACTGATTCTGCTGGCACAGATGCGTCG
TATTAGCCCGTTTAGCTGTCTGAAAGATCGTCATGATTTTGGT
TTTCCGCAAGAGGAATTTGATGGCAACCAGTTTCAGAAAGCA
CAGGCAATTAGCGTTCTGCATGAAATGATTCAGCAGACCTTTA
ACCTGTTCAGCACCAAAGATAGCAGCGCAGCATGGGATGAAA
GCCTGCTGGAAAAATTCTATACCGAACTGTATCAGCAGCTGAA
TGATCTGGAAGCATGTGTTATTCAAGAGGTTGGTGTTGAAGA
AACACCGCTGATGAATGTTGATAGTATTCTGGCCGTGAAAAA
ATACTTTCAGCGCATTACCCTGTATCTGACCGAAAAAAAGTAT
AGCCCGTGTGCATGGGAAGTTGTTCGTGCAGAAATTATGCGT

A G C T T T A G C A T T A G C A C C A A T C T G C A A G A A C G T C T G C G T C G C A
A A G A A T A A T A A

Figure S1c: Nucleotide sequence of the H-IFN construct in pET28a vector. Grey: His-tag; Red: cIFN sequence and Green: STOP codon.

Table S1: Sequence of nucleotides used in primer design. F, "forward"; R "reverse"; AT, annealing temperature.

Gene	Oligonucleotides	AT
h-ifn	F: 5´CGACCATGGGTCACCACCATCATCATCACTGTGATCTGCCGCAGACAC-3´<br>R: 5´CAGTCTCGAGTCACTATTCTTTGCGACGCAGACGTTC-3´	52 ºC
hd-ifn	F: 5´AGGGGATCCCTGAAAATACAGGTTTTCTTTACCGC -3´<br>R: 5´CGCCCATGGGCCATCATCACCACC-3´	55 ºC

Table S2. a: Composition of LB-agar medium.

Nutrients	Concentration (g/L)
Tryptone	10
Yeast Extract	5
NaCl	10
Agar	20

Table S2b: Composition of the complex medium of autoinduction. *Lactose was omitted in the groups that represent negative control.

Nutrients	Concentration (g/L)
Glycerol	5
Glucose	0,5
Lactose*	5,0
Yeast Extract	5,0
Phytone	10
KH_2PO_4	3,4
Na_2HPO_4	9
NH_4Cl	2,7
Na_2SO_4	0,7
$MgSO_4.7H_2O$	0,5
Ferric Citrate	100,8
$CoCl_2.6H_2O$	2,5
$MnCl_2.4H_2O$	15
$CuCl_2.2H_2O$	1,5
H_3BO_3	3
$Na_2MoO_4.2H_2O$	2,1
$Zn(CH_3COOH)_2.H_2O$	33,8
EDTA	14,1
Thiamine	45
Kanamycin	30

Table S2c: Composition of the chemically defined medium SDAB. *Lactose was omitted in the groups that represent negative control.

Nutrients	Concentration (g/L)
Glucose	2,94
Glycerol	11,07
Lactose*	7,6
KH_2PO_4	13,3
Citric Acid	1,55
$(NH_4)_2HPO_4$	4
$MgSO_4.7H_2O$	1,2
Ferric Citrate	100,8
$CoCl_2.6H_2O$	2,5
$MnCl_2.4H_2O$	15
$CuCl_2.2H_2O$	1,5
H_3BO_3	3
$Na_2MoO_4.2H_2O$	2,1
$Zn(CH_3COOH)_2.H_2O$	33,8
EDTA	14,1
Thiamine	45
Kanamycin	30

Table S2d: Composition of chemically defined medium HDF.

Nutrients	Concentration (g/L)
Glucose	-
Glycerol	10
KH_2PO_4	13,3
Citric Acid	1,7
$(NH_4)_2HPO_4$	4
$MgSO_4.H_2O$	1,2
Ferric Citrate	100,8
$CoCl_2.6H_2O$	2,5
$MnCl_2.4H_2O$	15
$CuCl_2.2H_2O$	1,5
H_3BO_3	3
$Na_2MoO_4.2H_2O$	2,1
$Zn(CH_3COOH)_2.H_2O$	33,8
EDTA	14,1
Thiamine	45
Kanamycin	30

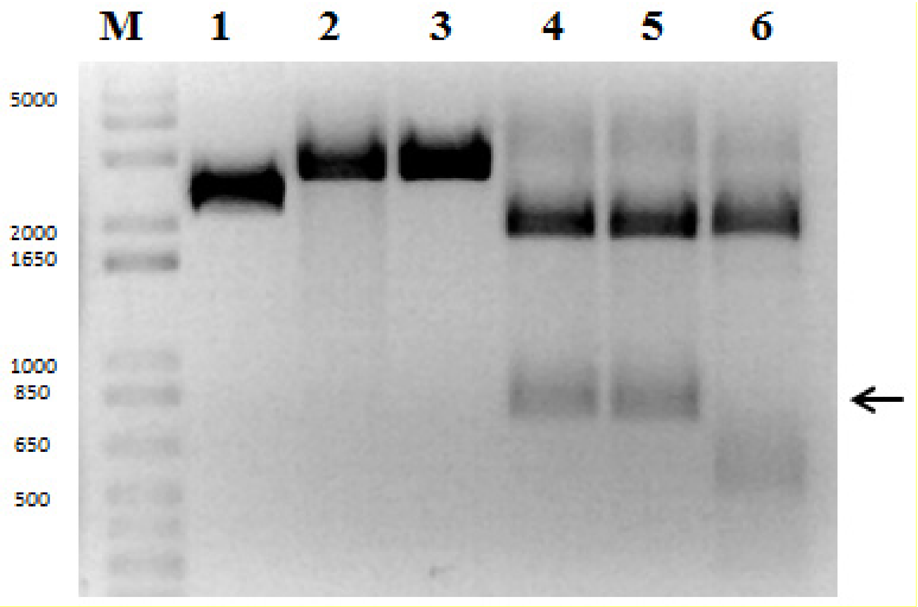

Figure S2: Restriction digest analysis for confirmation of the presence of the insert in the commercial plasmid. 1% agarose gel. (M) molecular marker 1kb DNA Ladder; (1) circular plasmid (2) linearized plasmid, digested with *Xho* I; (3) linearized, *Nco* I-digested plasmid; (4 and 5) plasmid digested with the enzymes *Xho* I and *Nco* I; (6) plasmid digested with *Xho* I, *Nco* I and *Bam* HI. The arrow indicates the released insert of 753 bp.

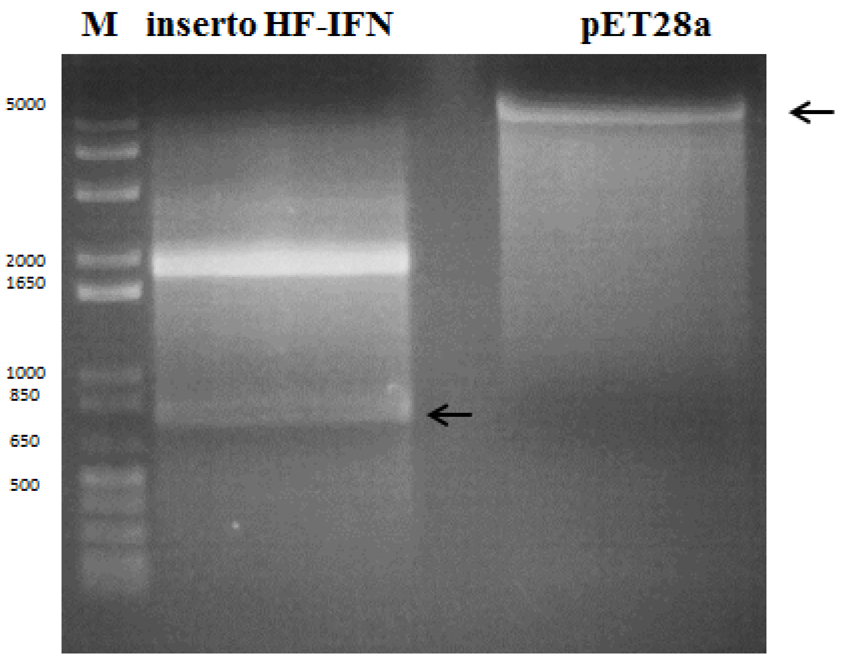

Figure S3: Preparative electrophoresis for HF-IFN insert and pET28a expression vector purification. 1% agarose gel. Plasmids were submitted to the enzymatic digestion protocol in the presence of the enzymes *Nco* I and *Xho* I. The fragments generated were used in the subsequent ligation reaction. (M) molecular marker 1kb DNA Ladder. The upper and lower arrows indicates the fragments that were purified: expression vector pET28a and HF-IFN insert, respectively.

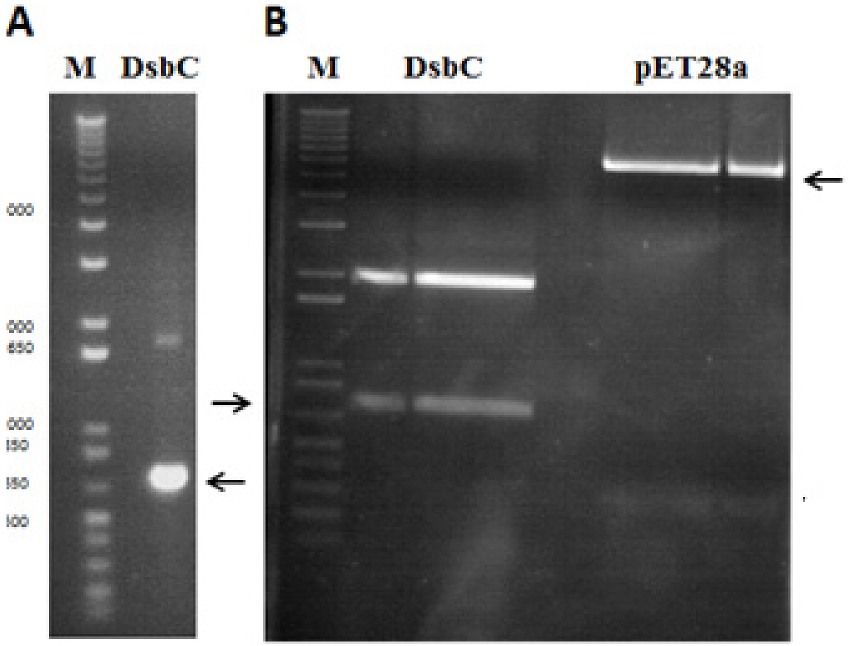

Figure S4: DsbC and pET28a cloning steps. 1% agarose gel. (A) Amplicon of the DsbC tag (648 nt). PCR was done using the commercial plasmid as DNA template; (B) preparative electrophoresis gel for purification of DsbC tag and plasmid digested with *Nco* I and *Bam* HI. The arrows indicate the fragments of interest. (M) molecular marker 1kb DNA Ladder.

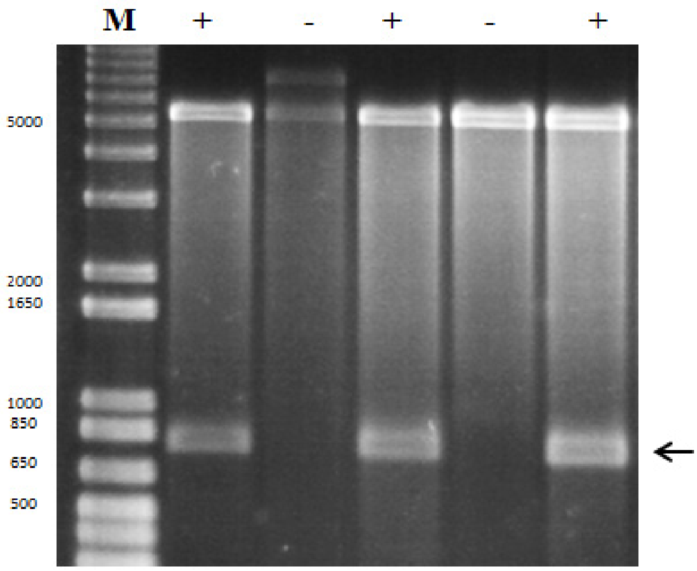

Figure S5: Selection of positive colonies for the HF-IFN insert. 1% agarose gel. Plasmids were submitted to the enzymatic digestion protocol in the presence of the enzymes *Nco* I and *Xho* I. The arrow indicates the insert released in the expected size, 753 nt. (M) molecular marker 1kb DNA Ladder. The + and - symbols respectively indicate bacterial colonies positive or negative for the HF-IFN insert.

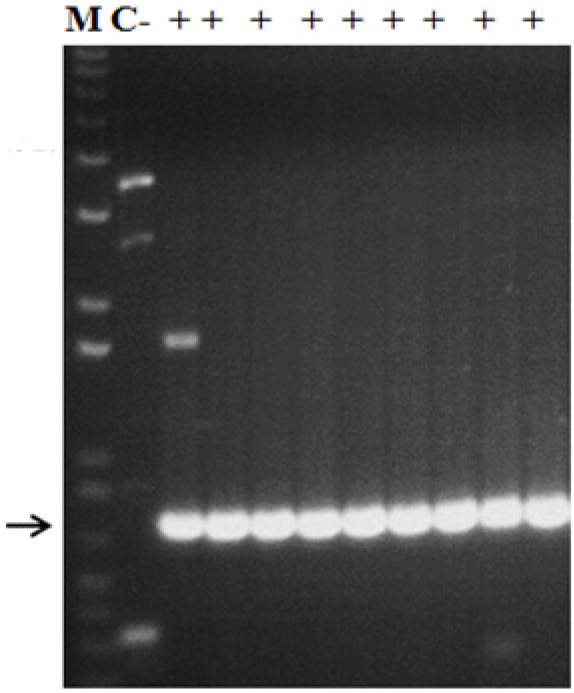

Figure S6: Positive *E. coli* colonies for plasmid pET28a-HD-IFN. 1% agarose gel. Bacterial colonies were submitted to PCR for detection of DsbC. The arrow indicates the fragment of interest. (M) molecular marker 1kb DNA Ladder. The + and - symbols indicate, respectively, bacterial colonies positive or negative for DsbC. C- indicates negative control.

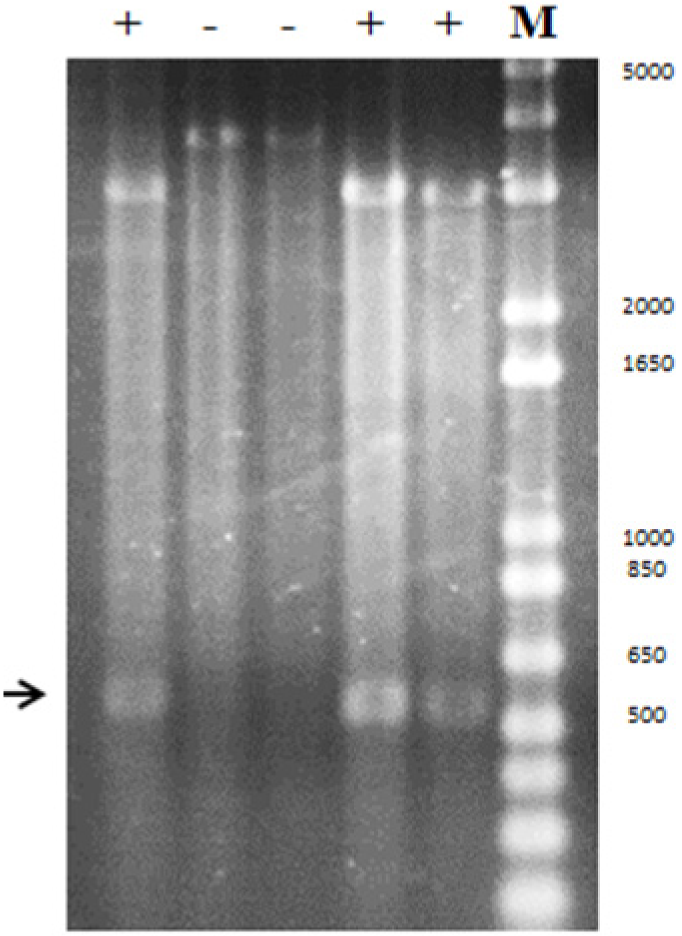

Figure S7: Electrophoresis gel for PCR-amplified H-IFN insert detection and selection of bacterial colonies. 1% agarose gel. Plasmids from bacterial colonies were submitted to the enzymatic digestion protocol in the presence of the enzymes *Nco* I and *Xho* I. The arrow indicates the presence insert in the expected size, 504 nt. (M) molecular marker 1kb DNA Ladder. The + and - symbols respectively indicate bacterial colonies positive or negative for the H-IFN insert, which does not have the Fh8 tag fused to the target protein.

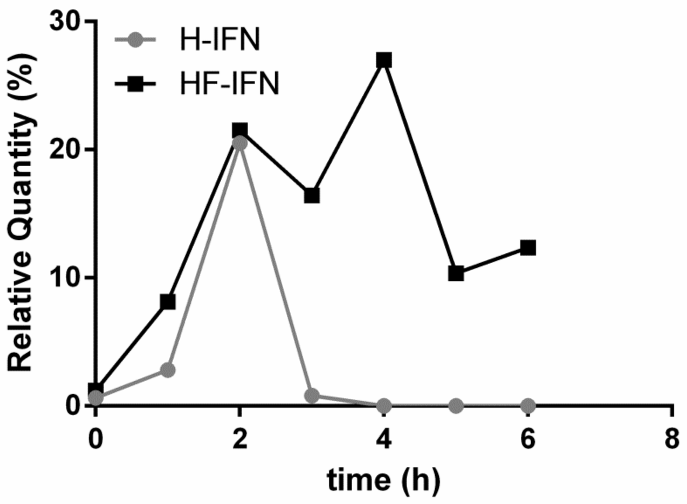

Figure S8: Quantification analysis of H- and HF-IFN in the soluble fraction. The relative amount of the target protein was determined by densitometry (Equation 1).

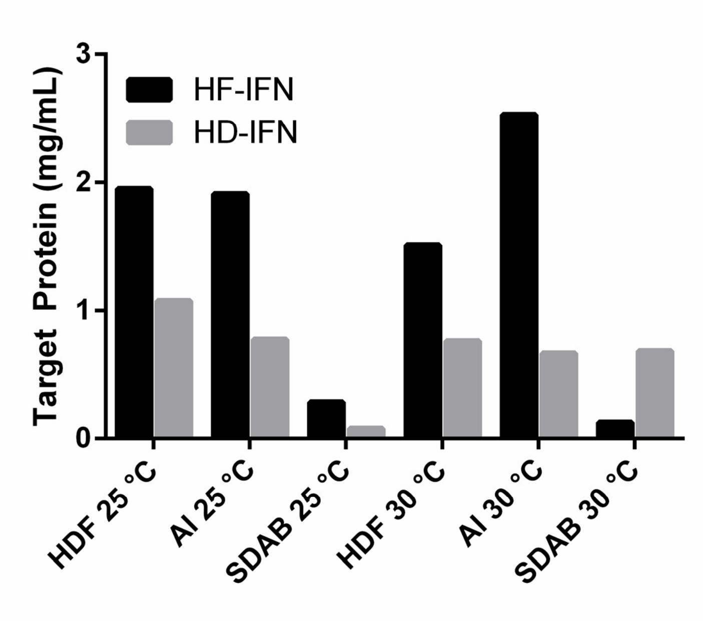

Figure S9. Comparison of HF-IFN and HD-IFN maximum production in different culture media and temperatures. The values reported refer to the maximum concentration of HF-IFN and HD-IFN reached in each culture media at 25 °C or 30 °C.

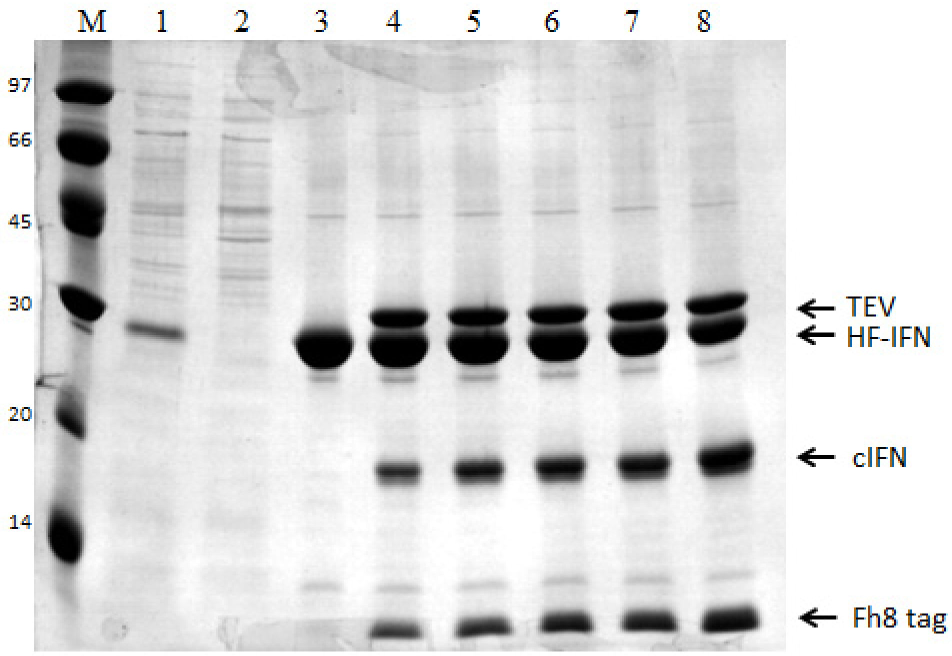

Figure S10. Enzymatic kinetics of HF-IFN hydrolysis in the presence of TEV. HF-IFN and TEV (5:1 proportion) were incubated in 20 mM Tris-HCl pH 8, containing 150 mM NaCl, 20% glycerol and 2 mM β-mercaptoethanol. (M) molecular weight standard (kDa); (1) non-hydrolyzed HF-

IFN; (2) non-induced BL21 (DE3) extract, negative control; (3) HF-IFN in the absence of TEV; (4) HF-IFN incubation with TEV for 1 hour; (5) HF-IFN incubation with TEV for 2 hour; (6) HF-IFN incubation with TEV for 3 hour; (7) HF-IFN incubation with TEV for 4 hour and (8) HF-IFN overnight enzymatic incubation with TEV. TEV has an approximate weight of 33 kDa; HF-IFN 28.8 kDa; cIFN 19.4 kDa and Fh8 tag 9.4 kDa. SDS-PAGE electrophoresis gel 15%.
